# Characterization of a Time Transfer Channel Between a Narrow-Band Transponder on a GEO Satellite and a Ground-Based Station

**DOI:** 10.3390/s26051515

**Published:** 2026-02-27

**Authors:** Ferran Valdes Crespi, Pol Barrull Costa, Angel Slavov, Matthias Weiß, Peter Knott

**Affiliations:** 1 Fraunhofer Institute for High Frequency Physics and Radar Techniques (FHR), Fraunhoferstr. 20, 53343 Wachtberg, Germany; pol.barrull.costa@fhr.fraunhofer.de (P.B.C.); angel.slavov@fhr.fraunhofer.de (A.S.); peter.knott@fhr.fraunhofer.de (P.K.); 2 Lehrstuhl Radar-Systemtechnik, Institut für Hochfrequenztechnik (IHF), RWTH Aachen University, 52062 Aachen, Germany; matthias.weiss@fhr.fraunhofer.de

**Keywords:** time transfer, medium propagation time delay measurement, signals-of-opportunity (Soops), netted radar, A-PNT, geostationary satellite QO-100

## Abstract

Time synchronization and positioning of bistatic radar transceivers is required to coordinate and meaningfully merge the measurements made between them. It simultaneously allows the radar transceivers to change their position throughout time. Despite their acknowledged vulnerabilities, Global Navigation Satellite Systems (GNSSs) are the preferred source for Positioning, Navigation and Timing (PNT) services. Because of these vulnerabilities however, research on possible signal sources to obtain alternative positioning, navigation and timing (A-PNT) is of interest. This present work proposes the use of a narrow-band transponder installed on a geostationary (GEO) satellite to be used as one anchor for a future time transfer. A channel calibration is made between the transceiver station and the chosen satellite. Diverse models are used to estimate the channel effects throughout the signal propagation path, estimate the time delay, and correct the measurements, accordingly. The available channel bandwidth on the proposed satellite is 2.7 kHz, limiting the accuracy of the time measurements. After integration of multiple pulses, a time accuracy of approximately 1 μs is obtained. The range measurements are compared against satellite positions propagated from publicly available two-line element sets (TLEs). The obtained results suggest that, after calibration, the expected accuracy and a good repeatability is obtained. Thus, making the QO-100 satellite a suitable anchor for the proposed technique.

## 1. Introduction

In general terms, the pace of actions of an electronic system is derived from its local oscillator (LO). Within the context of a bistatic radar system, time synchronization and positioning is a requirement for a distributed radar system, to correctly perform a radar measurement. In a previous publication [1], a detailed calibration was performed on the implemented measurement system based on software defined radios (SDRs). The transmission and reception of various waveforms were timed and performed. The present article intends to continue the development of this approach and serve to calibrate the signal propagation medium measured by a ground station using a transponder mounted on a GEO satellite. The future objective of the work is to determine the time offset between two radar transceivers. This is necessary to synchronize, i.e., reduce and subsequently maintain a time offset between a group of participating LOs of choice, to a level good enough for the resulting radar system to orderly trigger their respective measurement sequences.

Due to its ubiquitous availability, global coverage, and long-term time stability, the diverse GNSSs are generally the preferred source to obtain positioning, navigation, and timing services, despite their known vulnerabilities [2]. However, due to these vulnerabilities, the availability of such PNTs services can be disrupted or manipulated. Furthermore, in the short term, the timing service is not precise enough for a radar system mounted on moving platforms. For these reasons, the study of A-PNT sources is of interest [3,4]. This involves the identification and subsequent evaluation of suitable sources of signals of opportunity (SoOps).

Within the context of synchronization, diverse experimental time transfer methods that use optical signals, RF signals, or a combination of both methods have been shown to achieve remarkable levels of time accuracy [5,6]. In particular, optical-based time transfer systems exceed RF implementations over short baselines. However, these implementations are more sensitive to environmental effects, wherein RF implementations outperform optical implementations. A common limitation of the aforementioned experimental time transfer systems is usually the requirement of a static link, as well as the aforementioned constraint factor of the availability of line of sight (LOS).

Due to the availability of LOS, at the expense of long baselines and channel propagation effects, satellite-based references seem to be a suitable source of SoOps [7,8]. In ([9], Table 3.1), a list of ground- and satellite-based illuminators of opportunity such as FM, DAB-T, and Starlink can be found. A passive radar demonstrator using ground-based SoOps can be found in [10,11]. Often, the passive use of SoOpss is preferred, to avoid the use of an active transmission. However, the use of an active transmission allows one to obtain a time reference between when a signal was transmitted and the corresponding echo is received.

Without additional positioning information, a single one-way time transfer does not allow the medium propagation delay to be measured. A one-way time transfer implies that the receiver station receives a reference signal that was not initiated by it, while a two-way time transfer requires a transmitter or transceiver as the initiator and receiver of the transmission [12].

When comparing two clocks, the literature agrees that the most suitable time transfer method between these two fixed stations is a two-way time transfer throughout a satellite in common view ([13], Chapter 5) and [14]. This consists of two ground stations that bidirectionally transmits the encoded pulse-per second (PPS) of its LO periodically. The respective signals are relayed in turn by the satellite, using two separate channels of a certain bandwidth for their respective synchronization signals [15]. Both forward and reverse propagation paths may be approximated as if they were of equal value, and, therefore, cancel each other.

Since its invention, the Two-Way Satellite Time and Frequency Transfer (TWSTFT) procedure has been iteratively improved and it is used by the respective timekeeping laboratories worldwide to compare their respective primary time standards and for Coordinated Universal Time (UTC) calculation [16]. In Appendix F, a liaison and comparison between the standardized TWSTFT and the measurement method proposed in this work can be found.

Depending on the application, it is desired to use transmitted signals SoOpss from a satellite that do not require a specific stimulation from the receiver of interest. However, for time transfer purposes, a satellite equipped with a transponder has an advantage. A transponder responds to an incoming signal with, in principle, constant latency. Thus, introducing a certain additional time delay, it should be possible to compensate for it. However, the up- and down-links often use different carrier frequencies, and, therefore, the channel effects on the signal time delay on either direction need to be estimated.

If the relative position between the ground station and the satellite is estimated, the forward and reverse propagation path delays may be determined once all offsets and channel effects are taken into account. Furthermore, depending on the relative velocity and distance between the pair of platforms, the forward and reverse propagation paths are no longer symmetrical, and the geometric error becomes significant [17,18].

In addition to the signal-to-noise ratio (SNR) available on the received side, the type and modulation of the waveform used to carry out the time transfer will have the largest impact in terms of measurement accuracy. A trade-off lies in the fact that a signal that can accurately measure distance is inversely proportional to a signal that can accurately measure a frequency change, i.e., Doppler shift ([19], Chapter 11.4) and [20].

For the aforementioned reasons, a satellite located at GEO has been found to be suitable for this experimental characterization. Due to its low relative velocity with respect to a point on Earth, it serves for system calibration. It allows the contribution of different error sources to the measurement of time transfer to be quantified. The Qatar-OSCAR 100 (QO-100), with NORAD catalog number 43700, has been chosen.

The present article presents a single-station loop-back time-delay measurement. Such an intermediate step enables direct characterization as, although it does not provide a time transfer between independent clocks, it employs the same signal properties, correlation processing, and estimation algorithms as the proposed two-station architecture.

The rest of the article is organized as follows: in Section 2, the relative geometry of the experiment is explained, and, thereafter, in Section 3, the employed satellite position estimation is described. In Section 4 and Section 5, a synchronization signal is proposed, its parameters are thoroughly described, and a measurement error budget is established, respectively. In Section 6, Section 7 and Section 8, the experimental set-up, results, and discussion are presented. In Section 9, the article closes with a conclusion and an outlook for future work.

## 2. Geometry and Link Budget Between the Ground Station and the GEO Satellite

Across the parallels and meridians, the QO-100 is, with regard to a ground station, about 5° above the horizon for longitudes between 50° W and 100° E and 75° N and 75° S, respectively.

**Figure 1:**
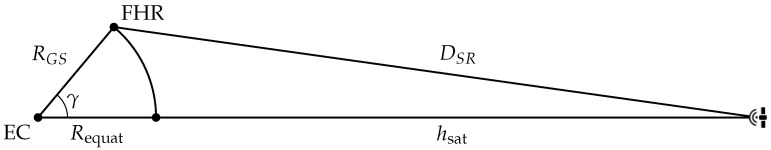
Diagram of the slant range SR between the ground station located at the FHR and the QO-100 GEO satellite. $R_{GS}$ represents the Earth radius, from the Earth’s Center (EC) to a point on the Earth’s surface, where the GS is located. γ is the geocentric angle between the GS and the satellite projection on the Earth equator.

In Figure 1, a representation of the relative geometry between the ground station and the GEO satellite is depicted. The maximum possible distances, defined by the northernmost or southernmost position, as well as the easternmost and westernmost, are the ground station where LOS is present with the satellite. That is, assuming that no negative elevation angle is possible, given the most unfavorable scenario, the tangent line to Earth, i.e., Edge of Coverage (EoC), is written thus

(1) $$D_{max}=\sqrt{{\left(h+R_{E}\right)}^{2}-R_{E}^{2}}$$

where, $R_{E}$ is the radius of the Earth at a given latitude. Taking into account that the Earth is an oblate spheroid, it is symmetric around the longitude north–south axis. The height $h_{sat}$ is the zenithal distance of the orbit of the satellite above the equator. The radius of Earth at the equator, when using the WGS84 model, further described in Appendix E, is 6378.137 km. The height of the satellite ideally is 35,786 km. This corresponds to a maximum distance of $D_{max}=\sqrt{{\left(35786+6378.137\right)}^{2}-6378.137^{2}}\approx 41679$ km.

Beyond this boundary case, for any arbitrary elevation angle, the slant range between the ground station and the satellite, adapted from the law of cosines is

(2) $$D_{SR}=\sqrt{R^{2}+{\left(R+h\right)}^{2}-2\cdot R\cdot \left(R+h\right)\cdot \cos\left(\gamma \right)}$$

with γ being the central angle defined according to the general spherical law of cosines as,

(3) $$\cos\left(\gamma \right)=\sin\left(\phi_{gs}\right)\cdot \sin\left(\phi_{sat}\right)+\cos\left(\phi_{gs}\right)\cdot \cos\left(\phi_{sat}\right)\cdot \cos\left(\lambda_{gs}-\lambda_{sat}\right)$$

ϕ:Latitudeλ:Longitude

where for a GEO satellite, it simplifies because $\phi_{sat}$ is zero,

(4) $$\cos\left(\gamma \right)=\cos\left(\phi_{gs}\right)\cdot \cos\left(\lambda_{gs}-\lambda_{sat}\right)$$

The measurements were carried out at the Fraunhofer Institute for High Frequency Physics and Radar Techniques (FHR) in Berkum, Germany. The coordinates for the Ground Station (GS) are 50.617° N, 7.132° E. The satellite is positioned relative to the GS, at an elevation of 29.4° and 153.1° in azimuth with respect of magnetic north. The height of the geodetic altitude *h*, above the reference ellipsoid of the WGS84 model, using the interpolated Earth gravity model EGM2008 is

(5) h=N+H

where *N* is the geoid undulation, *H* is the height above the mean sea level measured on-site with a calibrated GNSS receiver and are of values 47.5941 m and 262.4 m, respectively. Thus, h≈310 m. It can then be added to the location-dependent radii of the ellipsoid, $R_{E}$, the ellipsoidal height *h* of 310 meters for a total of $R_{gs}=R_{E}+0.310=6365.711$ km and compute the slant range $D_{SR}$, substituting Equation (4) in Equation (2)

$$\cos\left(\gamma \right)=\cos\left(50.617^{{}^{\circ}}\right)\cdot \cos\left(7.132^{{}^{\circ}}-25.8^{{}^{\circ}}\right)$$

$$D_{SR}=\sqrt{R_{gs}^{2}+{\left(R_{equat}+h_{sat}\right)}^{2}-2\cdot R_{gs}\cdot \left(R_{equat}+h_{sat}\right)\cdot \cos\left(\gamma \right)}$$

$$D_{SR}=\sqrt{{\left(6365.715\right)}^{2}+{\left(6378.137+35782.74\right)}^{2}-2\cdot \left(6365.715\right)\cdot \left(6378.137+35782.74\right)\cdot \cos\left(\gamma \right)}$$

An angle γ of ≈53.05° and a slant range of 38,670.402 km are obtained. Therefore, the signal round-trip time should be approximately 0.2579450254 s.

### Link Budget

It is of interest to use the narrow-band transponder on-board the QO-100 satellite as a relaying station. According to the available documentation from various amateur radio associations, the transponder is a triple conversion superheterodyne receiver. It uses two different LOs to up-shift the incoming signal from the up-link to the down-link channel. It has a bandwidth of 250 kHz, and the up-link and down-link frequencies are between 2400.05 and 2400.30 MHz and from 10,489.55 to 10,489.80 MHz, respectively. A higher frequency has a higher propagation path loss. Additionally, in the up-link a loss of 3 dB appears due to the antenna employed at the ground station being vertically polarized whereas the QO-100 satellite uses a right-hand circularly polarized antenna. This mismatch causes the antennas to capture only half of the incoming power.

The channel path loss can be estimated using the Friis transmission Equation [21]

(6) $$L_{FSPL}=20\log\left(d\right)+20log\left(f_{c}\right)+20log\left(\frac{4\pi }{c}\right)\;\left[\mathrm{dBm}\right]$$

where *d* is the distance [m], $f_{c}$ the carrier frequency [Hz], and *c* is the propagation velocity of the signal [ms] throughout the transmission medium. In Table 1, the free-space path loss (FSPL) is calculated for the expected and maximum possible slant ranges.

The power received by the ground station, $P_{R}^{GS}$, needs to account for the loss of up-link to determine the power received at the input of the satellite receiver with

(7) $$P_{R}^{SAT}=EIRP^{GS}-L_{FSPL}^{UL}-L_{ATM}^{UL}+G_{ANT_{RX}}^{SAT}+L_{POL}^{UL}$$

where the Equivalent Isotropic Radiated Power (EIRP) includes the transmitted power, cable losses, and antenna gain, $EIRP^{GS}=P_{TX}^{GS}-L_{GS}^{CABLE_{TX}}+G_{GS}^{ANT_{TX}}$. The satellite amplifies the received signal, but also introduces losses, modeled as $G_{CONV}^{SAT}$$=G_{XPDR}^{SAT}-L_{INT}^{SAT}$. The power the spacecraft retransmits down to Earth depends on this conversion, the power received, and the gain of the transmitting antenna. It’s EIRP is

(8) $$EIRP^{SAT}=P_{R}^{SAT}+G_{CONV}^{SAT}+G_{ANT_{TX}}^{SAT}$$

Similarly to the up-link in Equation (7), the power received at the ground station is

(9) $$P_{R}^{GS}=EIRP^{SAT}-L_{FSPL}^{DL}-L_{ATM}^{DL}+G_{ANT_{RX}}^{GS}+G_{LNB}^{GS}-L_{CABLE_{RX}}^{GS}$$

substituting the afore-explained expressions,

$$P_{R}^{GS}=P_{R}^{SAT}+G_{CONV}^{SAT}-L_{FSPL}^{DL}-L_{ATM}^{DL}+G_{ANT_{RX}}^{GS}+G_{LNB}^{GS}-L_{CABLE_{RX}}^{GS}=$$

$$=EIRP^{GS}-L_{FSPL}^{UL}-L_{ATM}^{UL}+G_{ANT_{RX}}^{SAT}+G_{CONV}^{SAT}-L_{FSPL}^{DL}-L_{ATM}^{DL}+G_{ANT_{RX}}^{GS}+G_{LNB}^{GS}-L_{CABLE_{RX}}^{GS}=$$

$$=P_{TX}^{GS}-L_{GS}^{CABLE_{TX}}+G_{GS}^{ANT_{TX}}$$

$$-L_{FSPL}^{UL}-L_{ATM}^{UL}+G_{ANT_{RX}}^{SAT}+G_{CONV}^{SAT}-L_{FSPL}^{DL}-L_{ATM}^{DL}+G_{ANT_{RX}}^{GS}+G_{LNB}^{GS}-L_{CABLE_{RX}}^{GS}$$

with the various parameters for the transceiver chain, listed in Table 2, that is a power received of $P_{R}^{GS}=38.25-115.81=-77.56$ dBm. The SNR is defined as the signal to noise ratio power, which is $SNR=P_{R}-P_{N}$ and $P_{N}=-174+10\log B+10\log\frac{T_{sys}}{290}+G_{LNB}-L_{CABLE_{RX}}$, at the input of the transceiver, where the first term, $T_{sys}$ is the system’s temperature calculated as $T_{ant}+\left(NF_{LNB}-1\right)\cdot T_{ant}$, B is the bandwidth in Hz, and NF is the noise figure of the reception chain, claimed to be 0.1 dB. This results in a noise power of $P_{N}=-174+34.31+0.1+57-2.2=-84.78$ dBm.

To account for atmospheric attenuation, an estimated density of 12 g of water per cubic meter, from ([22], Figure 1) and [23] has been used. It can be seen that the zenithal attenuation is approximately 0.04 dB for the lower transmission frequency and 0.1 dB for the higher receiving frequency. Using the flat-Earth approximation from [23] and ([24], Section 3.3), given the elevation angle, it results in 0.081 dB and 0.204 dB, respectively. Rain was not present during the measurements. Scintillation from ([22], Figure 1), is almost negligible, with the attenuation appearing only in the receiving band for 0.1 dB to 0.2 dB. It also specifies an attenuation due to clouds that could be present in the order of 0.1 dB for 2.4 GHz and 0.2 dB for 10.5 GHz carrier frequencies. That is a total estimate for atmospheric attenuation of approximately 0.2 dB and 0.6 dB for the up-link and down-link, respectively.

The SNR at the ground station is 7.22 dBm, which corresponds to the SNR during measurements.

## 3. Satellite Position Estimation

Diverse governmental institutions periodically measure and publish the positions of Earth orbiting satellites. The orbital parameters of the satellites are listed in TLE format at arbitrary time intervals. With the TLE information and by means of a propagator, the position of the satellite of interest may be estimated at a desired instant in time [25]. Of the different existing propagators based on the NORAD orbital model, Simplified Deep-Space Perturbations (SDP) is the most suitable to predict the position of GEO satellites, and has been used for the presented estimations. This model takes into account the influence of the moon, the sun, and some Earth harmonics [26].

The achievable position accuracy when using the SDP propagator depends on the difference between the experimental measurement instant and the TLE issue time. After a detailed analysis performed in [27], the radial and cross-track prediction errors remain relatively bounded compared to the in-track error. Although their analysis was performed using Global Positioning System (GPS) satellites, their results were intended to verify the accuracy of publicly available TLEs. This report shows that for propagation times less than 15 h, the estimation error for the radial and cross-track components are less than 3 km and less than 5 km for the in-track components. Similar results are reported in [28]. For certain satellites and for propagation times less than 24 h, the position uncertainty for all three axes seems to be close to 500 m [27,29]. Either error indicates the difference between the real position and the propagated position. A radial error is the difference in altitude, in-track is in the direction that the satellite moves, and cross-track is the deviation from its orbit.

### Measurement Validation

To estimate the position of the QO-100 satellite with respect to the location of the ground station at the measurement time, the orbit information is calculated from the most current TLE. In Table 3, the TLEs used to validate the experimental measurements are listed. The semi-major axis of the satellite orbit is calculated with the reported mean motion with

(10) $$A={\left(\frac{\mu }{n_{rad}^{2}}\right)}^{\frac{1}{3}}$$

where μ is the Earth gravitation coefficient of value 3.986004418·10−14[m3s2] and $n_{rad}$ is the mean motion in [rads].

**Figure 2:**
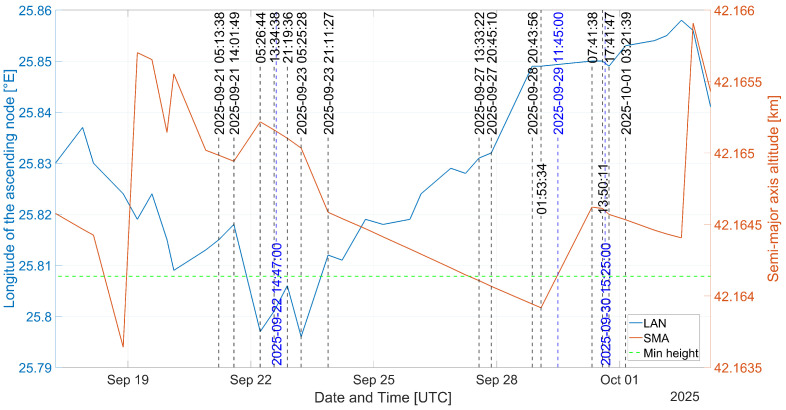
TLE for the GEO satellite QO-100 from 22, 29, and 30 September 2025. The vertical blue dashed traces indicate the date and time when a measurement was made. The vertical black dashed lines represent the propagated TLEs, which were used for the calculation to increase the satellite position estimation accuracy, following the method proposed by [27]. The labels where only a time of day is written correspond to the same date as when the experimental measurement is made. Within the shown time period, the maximum Apogee and minimum Perigee heights are 42,165.737 km and 41,163.637 km, respectively.

In Figure 2, the estimated position of the QO-100 satellite are plotted. The orange trace represents the height of the satellite above the Earth’s equator. The horizontal green dashed line represents the ideal GEO height. The vertical dashed blue and black traces represent the time when a measurement or a TLE was made or issued, respectively. The blue trace represents the elevation of the satellite with regards of the GS.

The TLEs information is encoded using a True Mean Equinox Frame (TEME) coordinate system. To calculate the slant range at any point on the Earth’s surface, a coordinate conversion to Earth-centered, Earth-fixed (ECEF) is required. With the calculated coordinates over time, a vector ${\vec{r}}_{sat}\left(t\right)$ is obtained and the slant range ${\vec{r}}_{gs}$ is calculated. The time delay is obtained with

(11) $${\hat{\tau }}_{geom}=\frac{\left|\right|{\vec{r}}_{sat}-{\vec{r}}_{gs}\left|\right|}{c}$$

where *c* is the medium propagation speed. It allows the estimated positions of the propagator ${\hat{\tau }}_{geom}$ and the experimental measurements $\tau_{MEAS}$ to be compared.

## 4. Synchronization Signal Design

In general terms, the time accuracy of a radar measurement depends both on the length of the transmitted signal and the SNR available at the receiver. The range resolution is inversely proportional to the transmitted pulse length. A shorter pulse length contains less energy and, due to path loss, has a lower potential range. Spectrum spread techniques are used to increase the effective bandwidth of the transmitted waveform. The techniques may be classified within the following three groups: chip sequence, frequency hopping, and frequency sweep. In all three variants, the energy of the resulting signal is distributed throughout the resulting spectrum in a different manner, but in all cases a larger channel bandwidth is required for the transmission. In techniques based on Direct-Sequence Spread Spectrum (DSSS), the transmitted signal s(t) is the baseband signal p(t) with a period $T_{p}$ modulated with a sequence c(t) with a period $T_{c}$, which needs to be shorter than $T_{p}$. The processing gain is TpTc, and it determines the bandwidth required to transmit the signal. The sequence modulates the phase of the signal. The resulting signal spreads relatively uniformly and has a bandwidth of 1TC, occupying the entire selected band. When using the frequency hopping spread spectrum (FHSS) technique, the carrier frequency is switched over a pre-defined sequence. The transmitted signal is narrowband; the instantaneous energy is contained in each transmission, but on average it is spread among the n-hops of the sequence. When using a chirp spread spectrum (CSS) technique, the carrier frequency is linearly increased or decreased throughout a desired range, and the signal energy is spread over this bandwidth.

A method to improve the radar range resolution is the use of pulse compression, which is a pulsed variant of the CSS technique. The DSSS technique can also be used, by transmitting a known sequence such as a Barker code [30]. A Barker code is a sequence of ones and zeros that modulates each pulse with binary phase shift keying (BPSK). The maximum length of the declared Barker codes is a sequence of 13 values, and to further decrease the sidelobe level (SLL), nested Barker codes may be used. Other sequences with different auto correlation properties exist, but do not differ in the working principle. When using CSS, a chirp is transmitted instead, where the carrier frequency is linearly modulated throughout the duration of a pulse ([19], Chapter 11). The objective of such techniques result in obtaining a large peak-power with a low-sidelobed autocorrelation function, allowing to precisely calculate the time delay of an specific return, i.e., improve the range resolution.

Amplitude-modulated chirps have better Doppler resilience, range resolution, and peak sidelobe ratios (PSLRs) compared to nested Barker codes [31]. In the same metrics, frequency modulated (FM) linear chirps outperform amplitude modulated (AM) chirps, owing to their direct frequency modulation and overall higher time-bandwidth product. For this reason, nested Barker codes are also outperformed by linear FM chirps.

### 4.1. Transmitted LFM Signal

Despite the advantages that a single linear frequency modulation (LFM) waveform has, a slight shift in Doppler, referred to as Range–Doppler coupling, can easily shift the output of the peak of the matched filter in time [32]. Furthermore, in a distributed system, the diverse LOs are not correlated and can induce drift or Doppler effects from platform dynamics. To address this problem, a combination of up- and down-chirps are used.

The aforementioned analytic function consists of a sum of two identical linear chirps except for their slopes, where one increases frequency over time and the other decreases at the same rate. In base-band, the dual-chirp s(t) is thus contained in a $T_{PRI}$ long pulse p(t), described as:

(12) $$p\left(t\right)=\left\{\begin{array}{ccc} s_{↑}\left(t\right)+s_{↓}\left(t\right) & for & t\in [0,T_{tx})\\ 0 & for & t\in [T_{tx},T_{PRI}) \end{array}\right.$$

(13) $$s_{↑}\left(t\right)=A\cdot \exp\left[j2\pi \cdot \left(f_{0}\cdot t\;+\;\frac{\Delta f}{2T}\cdot t^{2}\right)\right]$$

(14) $$s_{↓}\left(t\right)=A\cdot \exp\left[j2\pi \cdot \left(f_{0}\cdot t\;-\;\frac{\Delta f}{2T}\cdot t^{2}\right)\right]$$

where $f_{0}$ is the lowest base-band frequency of the chirp, *T* is the duration of the chirp, and m=Δf/T is the instantaneous frequency, i.e., the slope. The resulting linear FM dual-chirp spans a bandwidth Δf of 2.7 kHz, to comply with the self-regulating amateur radio guidelines for the use of the QO-100 narrow band transponder [33]. The phase of a chirp is quadratic in time and ensures a linear increase in frequency from 1 kHz to 3.75 kHz. In Appendix A the complete derivation of the dual-chirp is made.

#### Sampling Rate

The possible sampling rate on the universal software radio peripheral (USRP) x310 is selected with a decimation rate of $2^{10}$ steps, derived from its master clock rate of 200 MHz. The selected sampling rate is 1.923 MHz, which overly satisfies the Nyquist criterion, because the highest frequency component of the signal has a maximum value of $f_{s}/2=961.5$ kHz. Oversampling provides better time resolution, i.e., it decreases the bin size. This results in a sharper peak during the matched filtering process. The sampling frequency $f_{s}$ used results in a time granularity of

$$\Delta t=\frac{1}{f_{s}}=\frac{1}{1.923\cdot 10^{6}}\approx 0.52\;\mu s$$

### 4.2. Matched Filtering and Correlation

In order to detect the return and estimate its range, a matched filter has been implemented. It is demonstrated to be the optimal linear filter to maximize the SNR in the presence of additive stochastic noise ([19], Chapter 10). It consists of correlating the received signal with a copy of the transmitted signal thus

(15) $$R_{sr}\left(\tau \right)=\int r^{*}\left(t\right)s\left(t+\tau \right)dt=\int s^{*}\left(t\right)r\left(t+\tau \right)dt$$

which is equivalent to convolving the unknown signal with a conjugated time reversed version of it,

(16) $$R_{sr}\left(\tau \right)=r^{*}\left(-t\right)*s\left(t\right)=s^{*}\left(-t\right)*r\left(t\right)$$

where $s^{*}\left(t\right)$ is the complex conjugate of s(t) and τ is the time delay. In theory, correlation is implemented by sliding one signal past the other and computing the integrated product. This is shown in Equation (15), where the output $R_{sr}\left(\tau \right)$ measures the similarity between r(t) and a copy of s(t) shifted by τ. If a pronounced peak in $|R_{sr}\left(\tau \right)|$ is found, this would indicate that the received signal contains a delayed replica of the transmit signal at delay τ.

Since the transmitter and receiver were synchronized, it is not necessary to account for any unknown timestamp or starting offset. This allows the correlation to be directly calculated and the delay τ in relation to the start time of transmission to be interpreted.

Although s(t) and r(t) are continuous-time analog functions, once discretized, they are represented as discretized digital arrays by a sample-and-hold and analog-to-digital converter (ADC). The discrete cross-correlation is thus required,

(17) $$R_{sr}\left[\tau \right]=\sum_{n=0}^{N-1}s^{*}\left[n\right]\cdot r\left[n+\tau \right]$$

it can also be calculated with

(18) $$R_{sr}\left[\tau \right]=IFFT\left\{FFT\left\{s\left[n\right]\right\}\cdot FFT\left\{r^{*}\left[-n\right]\right\}\right\}$$

where τ is the time delay in samples instead of the time delay, and can be converted back with the relation:

(19) $$t=\tau \cdot \Delta t=\frac{\tau }{f_{S}}$$

For the present work, the complete five-pulse transmitted signal is compared with the entire received time series sampled during a five second period.

### 4.3. Ambiguity Function

The ambiguity function of a signal gives a characterization of the synchronization signal in terms of both its Doppler and delay resolution capabilities ([19], Chapter 11.4). It is described as

(20) $$X\left(\tau ,f_{D}\right)=\int_{-\infty }^{\infty }s\left(t\right)\cdot s^{*}\left(t-\tau \right)\cdot e^{-j2\pi f_{D}t}dt$$

where τ and $f_{D}$ are the time delay and Doppler shift, respectively. A time-dependent phase shift is added to the expression of the matched filter. Due to noise, the received signal is never exactly equal to the delayed attenuated and frequency shifted version of s(t). When a clear peak is found, the function presents a high correlation with the one sent for a certain delay and a certain Doppler $\left(\tau ,f_{D}\right)$. For the sake of completion, in Appendix C the full derivation of the Ambiguity Function (AF) can be found.

**Figure 3:**
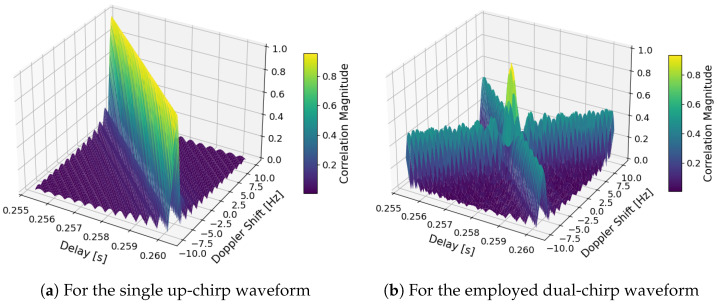
Computed AF of the proposed synchronization signal displaced in time at the expected signal propagation time delay.

**Figure 4:**
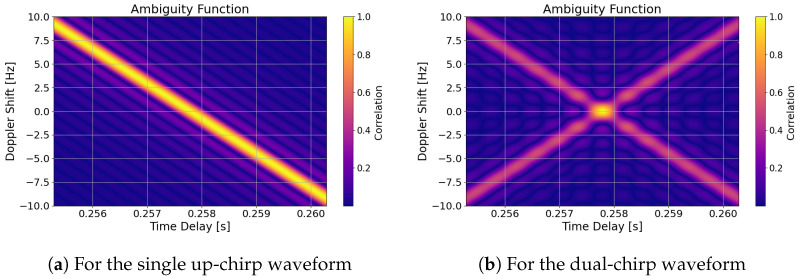
AF displaced in time to the expected return time of a 2.7 kHz bandwidth and 0.73 s long signal.

The AF of the proposed dual-chirp as described in Section 4.1, and depicted in Figure 3b and Figure 4b, exists only for |τ|≤T under the following expression,

(21) $$X_{D}\left(\tau ,f_{D}\right)=X_{↑↑}\left(\tau ,f_{D}\right)+X_{↓↓}\left(\tau ,f_{D}\right)+X_{↑↓}\left(\tau ,f_{D}\right)+X_{↓↑}\left(\tau ,f_{D}\right)$$

where,

$$X_{↑↑}\left(\tau ,f_{D}\right)={\left|A\right|}^{2}\cdot e^{j2\pi [f_{o}\tau -\frac{1}{2}m\tau^{2}]}\cdot e^{-j2\pi \left[\frac{T+\tau }{2}\right]\left[m\tau -f_{D}\right]}\cdot \left[1-\frac{\left|\tau \right|}{T}\right]\cdot \frac{sin(\pi T\left[m\tau -f_{D}\right]\left[1-\frac{\left|\tau \right|}{T}\right])}{\pi T\left[m\tau -f_{D}\right]\left[1-\frac{\left|\tau \right|}{T}\right]}$$

$$X_{↓↓}\left(\tau ,f_{D}\right)={\left|A\right|}^{2}\cdot e^{j2\pi [f_{o}\tau +\frac{1}{2}m\tau^{2}]}\cdot e^{-j2\pi \left[\frac{T+\tau }{2}\right]\left[-m\tau -f_{D}\right]}\cdot \left[1-\frac{\left|\tau \right|}{T}\right]\cdot \frac{sin(\pi T\left[-m\tau -f_{D}\right]\left[1-\frac{\left|\tau \right|}{T}\right])}{\pi T\left[-m\tau -f_{D}\right]\left[1-\frac{\left|\tau \right|}{T}\right]}$$

$$X_{↑↓}\left(\tau ,f_{D}\right)={\left|A\right|}^{2}\cdot e^{j2\pi [f_{o}\tau +\frac{1}{2}m\tau^{2}]}\cdot e^{-j2\pi [\frac{{\left(-m\tau -f_{D}\right)}^{2}}{4m}]}\cdot \frac{1}{\sqrt{2m}}\cdot {\left[Fr(u)\right]}_{u(a)}^{u(b)}$$

$$X_{↓↑}\left(\tau ,f_{D}\right)={\left|A\right|}^{2}\cdot e^{j2\pi [f_{o}\tau -\frac{1}{2}m\tau^{2}]}\cdot e^{j2\pi [\frac{{\left(m\tau -f_{D}\right)}^{2}}{4m}]}\cdot \frac{1}{\sqrt{2m}}\cdot {\left[Fr(v)\right]}_{v(a)}^{v(b)}$$

It has a limited resolution in both frequency and time. Such resolutions in time, Δτ, and in frequency, $\Delta f_{D}$, come from the main-lobe width, that is, given the first-null width, and as shown in Figure 3,

(22) $$\Delta \tau \approx \frac{1}{B}\;\;\Delta f_{D}\approx \frac{1}{T}$$

In Figure 3a,b, the ambiguity functions for a single and a dual LFM pulse, i.e., chirp, are plotted in 3D. In Figure 4a,b, the same ambiguity functions are plotted in 2D.

The ambiguity function of a single LFM pulse is skewed in its 2D plane. As mentioned previously, this Range–Doppler coupling can induce a shift in the matched filter in time due to a frequency, not Doppler solely, reference miss-match. For the single chirp case, the waveform cannot discern if the reception is not aligned in frequency with the transmission. As it presents an equal maximum value along the Range–Doppler line, as shown in Figure 4. Its delay bias Δτ caused by Doppler is dependent on the chirp slope *m*, and is approximately

(23) $$\Delta \tau \approx \frac{f_{D}}{m}=\frac{f_{D}\cdot T}{\Delta f}$$

It can be seen how a linear chirp offsets in delay estimation proportionally to its length and inversely proportionally to its bandwidth. For example, a 10 Hz shift contributes to $2.7\bar{037}$ ms, that is ∼810.55 km for the 2.7 kHz-wide signal employed or 73 μs or ∼21.89 km if the signal had a bandwidth of 100 kHz. However, in Figure 3b the autocorrelation function of the dual-chirp shows, when compared to a regular up- and down-chirp, a main to side lobe gain of 3 dB. The width of the main lobe can be adjusted to adjust the accuracy, where its ambiguity depends on the area of it.

## 5. Measurement Accuracy and Error Contributions

The discretization process introduces an uncertainty because the sampling instant has an unknown time offset with respect to the return signal time of arrival. The sampling time reference depends on Δt=1fs, and for the selected sampling frequency is ≈520.02 ns, which provides a range resolution $\Delta R_{sample}=c\;/\;2\cdot f_{s}$ of ≈77.94 m. However, because the bandwidth used for the current measurements is 2.7 kHz, the sampling rate suffices.

The theoretical accuracy of a radar measurement is dependent on the RMS extent of its spectrum S(f) [20] and it has been shown by Cook [34] that for a large enough time-bandwidth product, the spectrum of a LFM pulse approximates that of a rectangular pulse, yielding a range accuracy of

(24) $$\delta T_{r}=\frac{\sqrt{3}}{\pi \cdot B\cdot \sqrt{2\cdot E/N_{o}}}$$

In Appendix D, the full derivation of the time-delay error is made. For the simultaneous up- and down-chirps, for large BT, the cross-energy term is negligible (the Fresnel integral falls at a $O(BT^{-1})$ rate), therefore, the following approximation is made $E_{D}\approx E_{↑↑}+E_{↓↓}=|A_{↑}{|}^{2}T+{\left|A_{↓}\right|}^{2}T$. If the amplitude of the chirp, *A*, were to be equal for $A_{↑}$ and for $A_{↓}$, the normalized spectrum, and thus β, remains unchanged and thus the Root Mean Square (RMS) error decreases,

(25) $$\delta T_{r}^{\left(↑↓\right)}=\frac{1}{\beta \sqrt{2\cdot E_{↑↓}/N_{o}}}\approx \frac{1}{\beta \sqrt{4\cdot {\left|A\right|}^{2}\cdot T/N_{o}}}=\frac{1}{\sqrt{2}}\delta T_{r}^{\left(↑\right)}$$

However, the total energy is fixed, and each chirp’s component amplitude is halved, $A=\frac{1}{2}A_{↑}=\frac{1}{2}A_{↓}$. The time error is the same as in the single up-chirp case and the benefit solely remains on the previously discussed range-Doppler decoupling, not decreasing $\delta T_{r}$.

Although the channel bandwidth is recommended to be fixed at 2.7 kHz, within the pilot signals it would be feasible to use a bandwidth of up to 100 kHz. In Table 4, an overview of the achievable time accuracy and for diverse SNRs and bandwidth values is made. Because a sequence of chirps is made, an increase in the measurement accuracy is achieved by coherently integrating several pulses. The time-bandwidth product τB directly determines the achievable accuracy [20,35]. The resulting accuracy is calculated with $SNR_{CG}\;\left[\mathrm{dB}\right]=SNR_{per-pulse}+G_{PC}$, where the pulse compression gain is defined as $G_{PC}=10\log_{10}\left(\tau B\right)$, and in column $\delta T_{r,CG}$ from Table 4, its value is shown.

### 5.1. Measurement Error Budget

The measurement accuracy is composed of both the time dilation error contributor estimation [36] and the timing error and range resolution.

In order to quantify the contributions to the measurement uncertainty from the different sources and simultaneously validate the results, an additive two-way error budget estimation is calculated with

(26) $${\hat{\tau }}_{meas}TW=\delta_{xpdr}\pm \frac{{\hat{\delta }}_{time}}{2}+{\hat{\delta }}_{ion}UD+{\hat{\delta }}_{tropo}UD+\delta_{sagnac}+\delta_{trans}UD+\delta_{chain}$$

$$=10\;\mu s\pm \frac{1.295\;\mu s}{2}+8.664\;\mathrm{ns}+33.18\;\mathrm{ns}+2.406\;\mathrm{ps}+0.015\;\mathrm{ps}+36.692\;\mathrm{ns}$$

$${\hat{\tau }}_{meas}TW\approx 10.72603842\;\mu s\;\;\Delta R_{TW}\approx 3.21558542\;\mathrm{km}$$

where, $\delta_{xpdr}$ is the latency time of the transponder on-board the satellite, which seems to be of a transparent-type transponder. The incoming signal is not demodulated nor altered, it is frequency up-converted and re-transmitted ([37], Chapter 7). Its latency should be deterministic and constant; nevertheless, the schematic of the QO-100 transponder is not available in the open literature. Reference latency values for a transparent transponder are approximately 10μs ([38], Section 2.5.3A). Assuming such a delay, it would approximate an error uncertainty of approximately 3 km. ${\hat{\delta }}_{time}$ is the accuracy value obtained when an SNR of at least 8 dB is available, as listed in Table 4. Out of the different layers that compose the Earth’s atmosphere, the most significant contributors when estimating the time delay are the ionosphere and troposphere, $\delta_{ion}$ and ${\hat{\delta }}_{tropo}$, respectively. They vary depending on the frequency, angle of elevation, and slant range, and their effects are estimated from ITU-models. During the two-way time transfer, the forward and reverse propagation paths change its length, and therefore lead to a certain degree of uncertainty. This phenomenon is known as the Sagnac effect [39]. Furthermore, latencies and nonlinearities of the transceiver measurement equipment, $\delta_{trans}$, have been taken into account. Noise and quantization errors when using an UBX-160 daughter board within the x310 USRP for a measurement interval of five seconds are 0.015 ps ([1], Section 5.1.3). Out of the diverse factors that affect the time delay, because besides the low-noise block (LNB) and reflector antenna, all measurement equipment is placed in a permanent temperature-controlled room, where the temperature variations throughout a measurement are less than three degrees Celsius, a room temperature model has not been implemented [40]. The RF chain also adds a delay $\delta_{chain}$, mainly due to the length of the reception and transmission RF cables, connecting the USRP, LNB, power amplifier, and antenna. Its additive lengths for either reception and transmission paths are 5 and 6 m, which correspond to 16.678 ns and to 20.014 ns, respectively.

### 5.2. Troposphere Time Delay

The troposphere spans approximately between the Earth’s surface and up to a mean value of about 40 km and its effect on RF signals is refractive (change in speed and direction) due to the amount of water vapor in combination with nitrogen and oxygen (dry gases). A larger part of the water vapor is below 4 km and may be found up to about 12 km. About 75% of the total amount of nitrogen and oxygen is contained at a mean height of 12.5 km. The upper layer of the troposphere is dry, which accounts for ∼90% of the tropospheric delay, and the lower wet, which depends on regional conditions ([41], Chapter 5).

The time delay that a signals exhibits due to the troposphere is described as the excessive path length in the vertical component, $L_{v}$, mapped out to the slant range direction ([42], Equation (19)),

(27) $$\Delta L_{v}\approx 0.00227\cdot P+f\left(T\right)\cdot H$$

where

$$f\left(T\right)=a\cdot 10^{bT}$$

with *T*, *P*, and *H* being the meteorological surface inputs measured, temperature in Celsius, pressure in hPa, and humidity in percentage, respectively. The constants *a* and *b* are provided by the ITU and are $7.3\cdot 10^{-4}$ and $2.35\cdot 10^{-2}$, in that order. Given at an average values at time of measurement of 1022 hPa, 14 °C, and 78% humidity, the excessive length computes to 2.32 m for the hydrostatic component and 0.121 m for the wet component. That is a total vertical excess length, $\Delta L_{v}$, of 2.441 m. The ITU states that for elevation angles greater than 20 degrees the following mapping, m(β), can be used for both hydrostatic and wet components ([42], Equation (28f)),

(28) $$m_{h}\left(\beta \right)=m_{w}\left(\beta \right)=\frac{1}{\sin\beta }$$

Meaning the total excess length added by the troposphere is,

(29) $$\Delta L\approx \Delta L_{v,h}\cdot m_{h}\left(\beta \right)+\Delta L_{v,w}\cdot m_{w}\left(\beta \right)$$

Computing to 4.726 m in the hydrostatic component and 0.247 m in the wet component, for a total of 4.973 m or 16.59 ns for one-way.

### 5.3. Ionosphere Time Delay

The ionosphere spans approximately between 60 and 1000 km above the Earth’s surface, and its different layers are: *D*, *E*, F1, and F2. The peak electron density occurs at layer F2 at a height between 250 and 400 km ([41], Chapter 5). Signals traveling throughout the Ionosphere are subdued to a dispersive group delay. This depends on the totalelectroncontent(TEC), and is inversely proportional to the carrier frequency in use. The ionospheric delay effect is

(30) $$I_{f}=\frac{40.3\cdot TEC}{f^{2}}$$

where TEC is the electron content, TEC(β) in TECU units [$10^{16}$electronsm3] throughout the propagation path that depends on the elevation angle, 40.3 is approximated proportionality constant in [m3s2], and *f* is the carrier frequency in use. The larger the carrier frequency, the lower the effect on time delay [43]. The TEC depends on ionization radiation such as UV or X-ray emissions originating from the Sun, the Sun incidence angle and thus the season in the long-term and time of day in the short-term, and geographical location, as well as disturbances to the Earth’s electromagnetic field. The TEC values range from 0 to 100 units and the variability of the TEC value depends on the time of the day, with the values during the day usually being larger than those during night time.

Because the up- and down-links operate at different carrier frequencies, the medium propagation effects have different error contributions for either propagation path. By using two different carrier frequencies at the down-link, the path difference may be calculated and nullified ([44], Chapter 7). When only one frequency is available, the estimated TEC value for an specific region and time of day, provided with an update rate of 15 min, should be used [45].

The TEC value in the vertical direction, i.e., at zenith is the shortest path and for a satellite located at GEO, the path length is longer and the resulting ionospheric delay is obtained by multiplying the zenith delay with the obliquity factor, right term in Equation (31), such that

(31) $$TEC\left(\beta \right)=TEC_{zenith}\cdot {\left[1-{\left(\frac{R_{E}\sin\beta }{R_{E}+h_{I}}\right)}^{2}\right]}^{-\frac{1}{2}}$$

where β is the elevation angle, $R_{E}$ is the average Earth radius and $h_{I}$ is the mean ionosphere height of ∼350 km ([41], Chapter 5.3). That is a contribution from the ionosphere of 2.47 m or 8.23 ns in the up-link and 13 cm or 0.434 ns in the down-link.

### 5.4. Extension and Shortening of the Propagation Path

The correction on the Sagnac effect, $\delta_{sagnac}$, accounts for the Earth’s rotation on transmission in a non-inertial reference frame. It is usually insignificant since it often does not contribute for more than tens of nanoseconds but is of importance in attempting long-baseline, especially east–west, nanosecond-accurate links.

(32) $$\delta_{sagnac}={\vec{\Omega }}_{E}\cdot \frac{\left({\vec{r}}_{gs}\times {\vec{r}}_{sat}\right)}{c^{2}}$$

where ${\vec{r}}_{gs}$ and ${\vec{r}}_{sat}$ are the ECEF positions of the ground station and the satellite, and ${\vec{\Omega }}_{E}$ the Earth’s angular velocity vector in ECEF, $\left(0,0,\Omega_{E}\right)$. Contrary to one-way time dissemination methods, in a perfect two-way time transfer geometry, the Sagnac effect is practically zero since the Sagnac up-link and down-link terms mostly cancel out.

(33) $$\left|\Delta t\right|\approx \frac{\Omega^{2}\cdot R_{E}^{2}}{c^{2}}\cdot \Delta t_{meas}$$

where the Earth’s rotation rate is 7.291151467·10−5[rads]. That is a residual time for the 5 s that a measurement lasts, $\Delta t_{meas}$ of 2.406ps.

## 6. Experimental Set-Up

**Figure 5:**
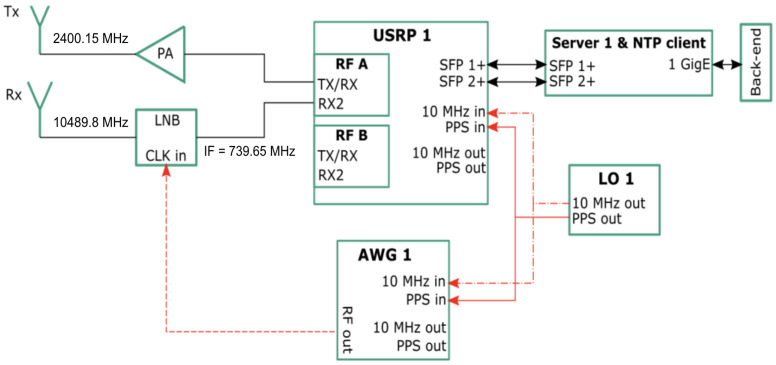
Experimental set-up for the time transfer with the QO-100 GEO satellite. It is composed of two separate paths for transmission and reception. The LNB clock signal is provided by the AWG. Both the AWG and USRP are referenced to the same free-running clock LO 1.

In Figure 5, the block diagram of the set-up used to carry-out the time transfer experiments is depicted. The transceiver part is composed of an USRP model x310 with two UBX-160 daughter boards installed in it [46]. As listed in Table 2, the transmission path comprises a 10 W amplifier with a max output of 38.25 dBm and a Schwarzbeck BBHA 9120 D horn antenna with a gain of 10.2 dBi. The reception path comprises a Goobay 67269 Ku-Band LNB, modified to accept an external LO reference signal to which its internal phase-locked loop (PLL) is synchronized, that provides a gain of 57 dB [47]. Both the LNB and the horn antenna are pointed towards their respective 85 cm reflector, with a gain of approximately 37.2 dBi. To ensure that the LNB is coherent with the USRP, a rubidium standard [48] is used. It is depicted as LO 1, and is set to free-run, serving as a reference for both the USRP and the arbitrary waveform generator (AWG). The AWG generates a reference signal of 25 MHz to set the LNB down-conversion factor to 390. The LNB LO is 9.75 GHz, downconverting from a RF of 10,489.505 Mhz to the intermediate frequency (IF) of 739.5 MHz.

Thus, the transceiver channels operate in a coherent manner [1]. A server computer with two SFP+ 10 Gbps interfaces, 64 GB of RAM, and an AMD epyc 7401p 24-core as CPU is used to control the USRP. The server computer runs on Linux OS and uses UHD version 4.0.0.0-240. The software part is programmed with GNU Radio and Python 3.11.

## 7. Experimental Results

Each measurement sequence consists of five successive simultaneous up-and-down pulsed LFM chirp signals with a pulse repetition interval (PRI) of one second. The duty cycle of the pulse is $T_{PRI}-T_{tx}=0.27$ s, which is just above the maximum expected time-of-flight between the satellite and the ground station. It is intended to maximize the duty-cycle as well as parallelly minimizing the error contribution induced by frequency drifts (either from Doppler or LO drift) by keeping the total transmission time as short as possible.

A set of ten batches of measurements per day was conducted. As described in Section Sampling Rate, with the sampling rate used over the total duration of transmission, it yields a total of 9,615,385 samples per measurement batch. The measures were started at $T_{0}$ and repeated with a time interval in between five and ten minutes. For the same period, the satellite orbit was calculated with the most recent TLE using a SDP propagator, as described in Section 3.

As a means of validation, the distance, $d_{MEAS}$, measured with the experimental set-up is compared with the propagated, $d_{SDP4}$, subtracting both the corresponding Δd is obtained. In Table 5, Table 6 and Table 7, the measurement results are listed. The time at which a measurement was made as well as the TLE age is noted in the respective caption as well. The age is the difference between the TLE issuing time and the measurement time. It can be seen that the difference between the measurements and the propagation output values remains practically invariant. In Table 8, descriptive statistics on all measurements are listed.

## 8. Discussion

In Figure 2, the estimated height of the QO-100 satellite, from the propagated positions at TLE issuing intervals, can be seen over time. It can be observed that during short periods of time, the satellite increases its height, probably due to a maneuver to reach the desired height of the operator. The spacing between TLE issues seems not to be constant.

To realize the experimental measurements, suitable weather conditions were used. Due to weather conditions, the measurements made on the 30 September span throughout 30 min instead of 1:15 and 1:30. Of the measurements performed, two batches were made while the satellite was descending and one batch was made whilst the satellite was increasing its height. Several TLEs were issued relatively close in time from the measurement instants for the case when the satellite was descending, that is, on 22 and 30 September. However, for the case where the satellite was ascending, no TLE was issued for a longer period of time, that is, on 29 September.

**Figure 6:**
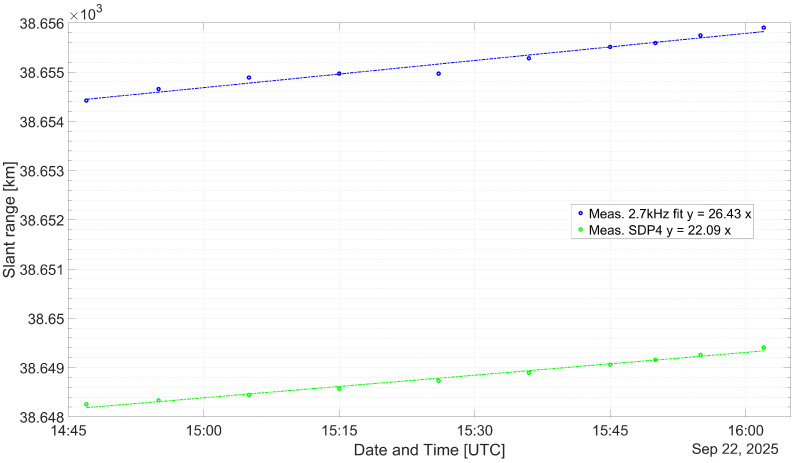
Comparison between the measured and propagated slant ranges for the QO-100 GEO satellite from 22 September 2025.

**Figure 7:**
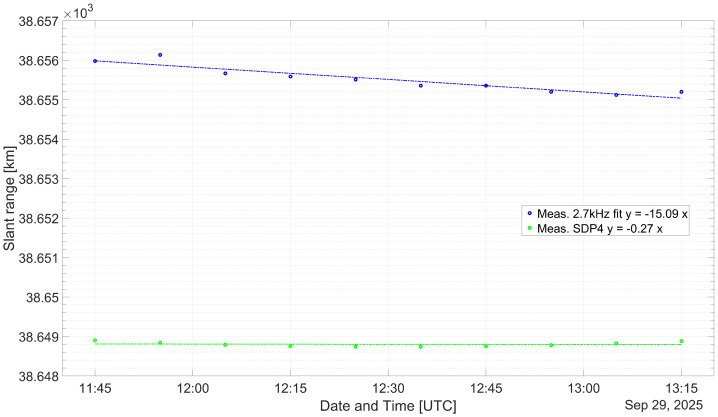
Comparison between the measured and propagated slant ranges for the QO-100 GEO satellite from 29 September 2025.

**Figure 8:**
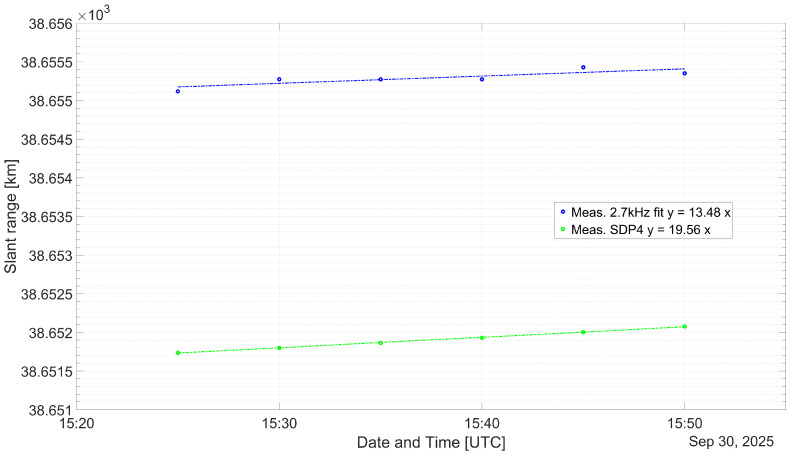
Comparison between the measured and propagated slant ranges for the QO-100 GEO satellite from 30 September 2025.

From [27], it is proposed that the publicly available TLEs have some embedded noise. In order to increase the accuracy when predicting a satellite position, propagating several TLEs issued close to the desired position time seems to be a commonly used method. It is suggested that for a short period of time, forward or reverse propagating a TLE is equally accurate. It was found that for the evaluated TLEs, the forward propagated had a better correlation and lower spread between themselves compared to when being reverse propagated. Nevertheless, during the ascending maneuver, the predicted satellite position diverged largely compared to the results obtained on 22 and 30 September. For this reason it was decided to choose the closest TLE issued to the measurement instant, as listed in Table 3, as well as the three previous and three next TLEs. In Figure 6, Figure 7 and Figure 8, the median of the propagated slant range for either of the TLEs is represented with a green dots.

Figure 6, Figure 7 and Figure 8 compare the estimated and measured satellite position for the corresponding measurement interval. In Table 5, Table 6 and Table 7, the measurements made with 2.7 kHz are represented by blue and red dots, respectively. The Column $d_{ADJUST}$ represents the measured slant range, taking into account the proposed measurement error budget from Equation (7). The Columns Δd and Δt are the range and corresponding time difference between the propagated and adjusted measurements. The trend lines have been overlapped with the measurements, as a dashed trace, showing in all cases a slope very similar to the propagated position of the respective TLEs.

In Section 5.1, an estimation of the error contribution from the transponder latency as well as the medium propagation effects is made. This error budget allows for the correction of the raw measurements. The transponder latency has been assumed according to the list of literature and is in the order of 10 μs. This latency is the largest contributor to the time offset, considering that it remains constant. The addition of all uncertainties adds-up to a combined error of 3.215 km. It results in an offset on the order of 6.5 km and 7 km for the measurements done on 22 and 29 September and of 3 km for the measurements carried out on 30 September. In all cases, the measured slant range is larger than the estimated satellite position. Although the slopes from the diverse linear regression are very similar, an additional time offset that is not taken into account seems to be present.

In Table 8, descriptive statistics from the measurements and propagated satellite positions are listed. The measurements and the estimated satellite positions made on 22 and 30 September are similar in terms of standard deviation, variance, and spread. The measurements made on 29 September are statistically comparable to the measurements made on 30 September; however, the estimated positions largely differ. The difference could be due to the ascending maneuver of the satellite. A TLE is issued at 1:53:34, which seems to be the point the ascending maneuver started. For this reason, the TLEs issued before and after the maneuver had to be used. The averaged positions between the considered TLEs to compare the measurements on 29 September are at different heights, and this may be the reason why the slope is close to zero, although the measurements suggest a loss of height instead of an increase. Other research suggest to discard measurements during an ascending maneuver. It was decided not to discard the measurements to allow a subsequent evaluation.

The standard deviation on the measurements made on 22 and 29 September suggests that the theoretical accuracy estimated for an SNR of approximately 8 dB, as listed in Table 4, is achieved. Although the SNR did not significantly improve for the measurements made on 30 September, it could be that just after finishing the ascension maneuver, the satellite is in a more stable situation. This is suggested by the low standard deviation and spread from both experimental measures and position estimation using the TLEs.

As detailed in Section 5, the accuracy of the time transfer largely depends on the τB product. Given a fixed τ, employing a 2.7 kHz of bandwidth, the satellite may be used as a lower precision common anchor. From Table 4, depending on the SNR available at the receiver, time accuracies better than 2 μs are expected. However, in the case of using a 100 kHz-wide signal and for low SNR values, a time accuracy better than 50 ns is to be expected, being comparable to a GNSS receiver. This level of time accuracy, is not sufficient for positioning of navigation purposes, as three more satellites would be required. However, this shows that a time transfer made with different transponders mounted on different satellites, or between several stations and this satellite, would lead to accurate results.

## 9. Conclusions and Future Work

The presented method requires the use of multiple anchors to provide a time reference to the participating nodes of the netted radar system. The current implementation employs a purposely adjusted radar waveform with a good trade-off between range and Doppler to measure the slant range between the ground station and the chosen satellite. The obtained slant range is compared with the estimated satellite positions, propagated from the seven closest TLEs, averaged.

Arguably, the orbit and position of satellites intended for GNSSs purposes are more precise than for satellites for other purposes. Furthermore, publicly available TLEs, as noted in various studies discussed in Section 3, have lower accuracy than what is in principle possible.

The use of a narrow-band transponder embedded in a GEO satellite, originally intended for voice communication, has been proposed as a suitable anchor to carry-out a two-way time transfer (TWTT) in the near future. The satellite used for the experimental measurements is the QO-100, which is the first satellite to include a radio amateur transponder at GEO stationary orbit.

A radar waveform to mitigate the effects caused by Doppler as a result of the relative movement between the satellite and the ground station has been proposed. Although the available bandwidth per user channel is small, i.e., 2.7 kHz, thus limiting the accuracy of the time transfer, successive measurements suggest repeatability and low spread. It should be possible to use a larger bandwidth without interfering with the pilot signals, as listed in Table 4. Thus, increasing the time accuracy from one microsecond to the order of low nanoseconds.

The position of the satellite is estimated using the seven closest publicly available TLEs to the measurement intervals. The measurements are validated by using the difference between the estimated and measured satellite positions. The measurement trends for the different measurements and orbit propagation suggest a good agreement and repeatability over a time span of three non-consecutive measurement days.

Further research will be carried-out to attempt to measure the latency of the QO-100 on-board transponder. This is intended to decrease the time offset in the measurement because it presumably accounts for the largest source of uncertainty. Once the transponder and other uncertainties in the time transfer are fully resolved, a time transfer may be initiated by the ground station and be received by a number of stations, including the initiating station. These stations may, in-turn, initiate a different time transfer as well.

## Figures and Tables

**Table 1:** Link budget between the ground station and the QO-100 satellite for the ideal and maximum slant range where the satellite would be still within LOS.

Carrier Freq. [MHz]	Range [km]	FSPL [dB]
2400.3	38,671	191.80
2400.3	41,679	192.45
10,489.8	38,671	204.61
10,489.8	41,679	205.26

**Table 2:** Parameters used to calculate the link budget. The values for the satellite antennas gains as well as its transponder amplification factor are estimated, based on the measured SNR at the ground station.

Parameter	Symbol	Value
Transmit Power (GS)	$P_{TX}^{GS}$	38.25 [dBm]
TX Cable Loss (GS)	$L_{GS}^{CABLE_{TX}}$	0.9 [dB]
TX Antenna Gain (GS)	$G_{GS}^{ANT_{TX}}$	10.2 [dBi]
Uplink FSPL	$L_{FSPL}^{UL}$	191.8 [dB]
Uplink Atmospheric Loss	$L_{ATM}^{UL}$	0.2 [dB]
Satellite RX Antenna Gain	$G_{ANT_{RX}}^{SAT}$	24 [dBi] (assumed)
Satellite TX Antenna Gain	$G_{ANT_{TX}}^{SAT}$	37 [dBi] (assumed)
Polarization miss-match loss	$L_{POL}^{UL}$	3 [dB]
Satellite Converter Gain	$G_{CONV}^{SAT}$	120.3 [dB] (assumed)
Downlink FSPL	$L_{FSPL}^{DL}$	204.61 [dB]
Downlink Atmospheric Loss	$L_{ATM}^{DL}$	0.6 [dB]
GS RX Antenna Gain	$G_{ANT_{RX}}^{GS}$	37.2 [dBi]
RX Cable Loss (GS)	$L_{CABLE_{RX}}^{GS}$	2.2 [dB]
LNB Gain (GS)	$G_{LNB}^{GS}$	87 [dB]

**Table 3:** TLEs used for the calculations and experimental verification. They were selected as the closest issued TLEs from the measurement time.

Epoch [UTC]	Meas Time [UTC]	Mean Motion *n* [revday]	Semi-Major Axis *A* [km]	Eccentricity *e*
2025-09-22 13:34:39	14:47	1.00270253	42,164.99	0.0001330
2025-09-29 01:53:34	11:45	1.00274691	42,163.75	0.0001474
2025-09-30 07:41:38	15:25	1.00272182	42,164.45	0.0001367

**Table 4:** Two-way theoretical measurement accuracy when using a dual chirp as measuring waveform. The values are calculated for diverse available SNR at the receiver, using two different signal bandwidths, without compression gain and with compression gain (CG). The last column is the range accuracy when using CG.

SNR [dB]	B [kHz]	$\delta T_{r}\;\left[\mu s\right]$	$\delta T_{r,\mathrm{CG}}\;\left[\mu s\right]$	δR[m]
5	2.7	81.20	1.8289	548.289
	100.0	2.19	0.0494	14.804
8	2.7	57.48	1.2948	388.158
	100.0	1.55	0.0350	10.480
12	2.7	36.26	0.8169	244.912
	100.0	0.98	0.0221	6.613
20	2.7	14.44	0.3252	97.501
	100.0	0.39	0.0088	2.632

**Table 5:** Measurement outline and comparison. Date: 22.09.2025, $T_{0}$ = 14:47:00 [UTC]. TLE’s age is 1:11.

Meas. [#]	BW [kHz]	Time [h:m]	$d_{\mathrm{SDP}4}$ [km]	$d_{\mathrm{MEAS}}$ [km]	$d_{\mathrm{AJUST}}$ [km]	Δd [km]	Δt [μs]
1	2.7	$T_{0}$	38,648.166	38,657.638	38,654.422	6.256	20.868
2	2.7	+8′	38,648.246	38,657.871	38,654.655	6.409	21.378
3	2.7	+18′	38,648.361	38,658.105	38,654.889	6.528	21.775
4	2.7	+28′	38,648.493	38,658.183	38,654.967	6.474	21.595
5	2.7	+39′	38,648.656	38,658.183	38,654.967	6.311	21.051
6	2.7	+49′	38,648.822	38,658.496	38,655.28	6.458	21.542
7	2.7	+58′	38,648.984	38,658.729	38,655.513	6.529	21.778
8	2.7	+1 h 03′	38,649.080	38,658.806	38,655.59	6.510	21.715
9	2.7	+1 h 08′	38,649.179	38,658.962	38,655.746	6.567	21.905
10	2.7	+1 h 15′	38,649.324	38,659.118	38,655.902	6.578	21.942

**Table 6:** Measurement outline and comparison. Date: 29.09.2025, $T_{0}$ = 11:45 [UTC]. Difference between epoch and measures is 9:52.

Meas. [#]	BW [kHz]	Time [h:m]	$d_{\mathrm{SDP}4}$ [km]	$d_{\mathrm{MEAS}}$ [km]	$d_{\mathrm{AJUST}}$ [km]	Δd [km]	Δt [μs]
1	2.7	$T_{0}$	38,648.657	38,659.196	38,655.98	7.323	24.427
2	2.7	+10′	38,648.592	38,659.352	38,656.136	7.544	25.164
3	2.7	+20′	38,648.543	38,658.884	38,655.668	7.125	23.766
4	2.7	+30′	38,648.510	38,658.806	38,655.59	7.080	23.616
5	2.7	+40′	38,648.492	38,658.729	38,655.513	7.021	23.420
6	2.7	+50′	38,648.490	38,658.573	38,655.357	6.867	22.906
7	2.7	+1 h 00′	38,648.504	38,658.573	38,655.357	6.853	22.859
8	2.7	+1 h 10′	38,648.535	38,658.417	38,655.201	6.666	22.235
9	2.7	+1 h 20′	38,648.580	38,658.339	38,655.123	6.543	21.825
10	2.7	+1 h 30′	38,648.642	38,658.417	38,655.201	6.559	21.878

**Table 7:** Measurement outline and comparison. Date: 30.09.2025, $T_{0}$ = 15:25 [UTC]. Difference between epoch and measures is 7:44.

Meas. [#]	BW [kHz]	Time [h:m]	$d_{\mathrm{SDP}4}$ [km]	$d_{\mathrm{MEAS}}$ [km]	$d_{\mathrm{AJUST}}$ [km]	Δd [km]	Δt [μs]
1	2.7	$T_{0}$	38,652.195	38,658.339	38,655.123	2.928	9.767
2	2.7	+5′	38,652.257	38,658.495	38,655.279	3.022	10.080
3	2.7	+10′	38,652.321	38,658.495	38,655.279	2.958	9.867
4	2.7	+15′	38,652.387	38,658.495	38,655.279	2.892	9.647
5	2.7	+20′	38,652.456	38,658.651	38,655.435	2.979	9.937
6	2.7	+25′	38,652.528	38,658.573	38,655.357	2.829	9.437

**Table 8:** Measurements statistics made for the corresponding single orbital propagation with an SDP4 and low-bandwidth measurements. According to Figure 2, the QO-100 satellite is descending on the 22-09 and on 30-09, and ascending on 29-09. The measurements made on 30-09 had a shorter time interval between measurements when compared to the other measurements.

Date	Measurement	σ [m]	$\sigma^{2}$	Median [km]	Spread [m]	Med (1st Diff)
22-09-25	TLE	408.1	166.5	38,648.739	1157.8	131.6
	med (TLEs)	405.1	164.1	38,648.811	1151.4	127.6
	2.7 kHz	489.3	239.3	38,658.339	1480.0	156.0
29-09-25	TLE	60.7	3.7	38,648.538	166.4	1.7
	med (TLEs)	58.7	3.4	38,648.786	161.8	−1.9
	2.7 kHz	338.6	114.6	38,658.651	1012.9	78.0
30-09-25	TLE	124.5	15.5	38,652.354	332.6	66.5
	med (TLEs)	127.1	16.2	38,651.900	339.5	67.9
	2.7 kHz	103.7	10.7	38,658.495	312.0	0.0

## Data Availability

The datasets presented in this article are not readily available because the measurements made within the scope of this work were funded by the the Fraunhofer institution, with grant Discover 40-10384. The data are part of an ongoing study. Requests to access the datasets should be directed to ferran.valdes.crespi@fhr.fraunhofer.de.

## Abbreviations

A-PNT	alternative positioning, navigation and timing
ADC	analog-to-digital converter
AF	Ambiguity Function
AM	amplitude modulated
AWG	arbitrary waveform generator
BPSK	binary phase shift keying
CDMA	code division multiple access
CSS	chirp spread spectrum
DSSS	Direct-Sequence Spread Spectrum
ECEF	Earth-centered, Earth-fixed
EGM	Earth Gravitational Model
EIRP	Equivalent Isotropic Radiated Power
EoC	Edge of Coverage
FHR	Fraunhofer Institute for High Frequency Physics and Radar Techniques
FHSS	frequency hopping spread spectrum
FM	frequency modulated
FSPL	free-space path loss
GEO	geostationary
GNSS	Global Navigation Satellite System
GPS	Global Positioning System
GS	Ground Station
IF	intermediate frequency
LFM	linear frequency modulation
LNB	low-noise block
LO	local oscillator
LOS	line of sight
NORAD	North American Aerospace Defense Command
PLL	phase-locked loop
PNT	Positioning, Navigation and Timing
PPS	pulse-per second
PRI	pulse repetition interval
PRN	pseudo-random-noise
PSLR	peak sidelobe ratio
QO-100	Qatar-OSCAR 100
RF	radio frequency
RMS	Root Mean Square
SDP	Simplified Deep-Space Perturbations
SDR	software defined radio
SLL	sidelobe level
SNR	signal-to-noise ratio
SoOps	signals of opportunity
TEC	total electron content
TEME	True Mean Equinox Frame
TLE	two-line element set
TWSTFT	Two-Way Satellite Time and Frequency Transfer
TWTT	two-way time transfer
USRP	universal software radio peripheral
UTC	Coordinated Universal Time
WGS84	World Geodetic System 1984

## Appendix A. Derivation of the Linear Chirp Formula

A linear chirp has a linearly varying frequency f(t), thus:

(A1) $$f\left(t\right)=mt+f_{0}$$

where $f_{0}$ is the starting frequency, and *m* is the chirp rate or slope at which the frequency will change over time, thus

(A2) $$m=\frac{f_{1}-f_{0}}{t_{1}-t_{0}}=\frac{\Delta f}{T}$$

with $f_{1}$ being the end frequency, and $t_{1}$ and $t_{0}$ the end and start times, respectively. Δf is the bandwidth of the chirp and *T* is the duration of the pulse.

Thus, by increasing or decreasing the phase ϕ(t) over time, the resulting signal is

(A3) $$s\left(t\right)=A\cdot e^{j\phi (t)t}$$

where *A* is an scaling factor and the instantaneous phase ϕ(t), is the integral of its instantaneous frequency over the period *t* for,

$$f\left(t\right)=\frac{1}{2\pi }\frac{d\phi (t)}{dt}$$

meaning,

(A4) $$\phi \left(t\right)=\phi_{0}+2\pi \int_{0}^{t}f\left(\tau \right)d\tau =\phi_{0}+2\pi \int_{0}^{t}\left(\frac{\Delta f}{T}\tau +fo\right)d\tau$$

(A5) $$\phi \left(t\right)=\phi_{0}+2\pi \cdot {\left[\frac{\Delta f}{2T}\cdot \tau^{2}+f_{0}\cdot \tau \right]}_{0}^{t}=\phi_{0}+2\pi \cdot \left(\frac{\Delta f}{2T}\cdot t^{2}+f_{0}\cdot t\right)$$

where $\phi_{0}$ is the initial phase at $t_{0}$. The phase has an exponent of second order on the time variable, achieving the requisite of linear growth with it, and it is therefore a quadratic-phase signal. The time-domain function for the phase in Equation (A5) is substituted-in Equation (A3). It results in a linear up- or down-chirp, whose width depends on the selected boundary frequencies $f_{0}$ and $f_{1}$ such

(A6) $$s\left(t\right)=A\cdot e^{j2\pi \cdot \left(\pm \frac{\left|\Delta f\right|}{2T}\cdot t^{2}+f_{0}\cdot t\right)}$$

the aforementioned expression is defined for all t∈(−∞,∞), however, the signal is transmitted over a finite duration *T*. That is, to properly model it, the signal s(t) is multiplied by a unity energy causal rectangular pulse, $p_{T}\left(t\right)$,

(A7) $$p_{T}\left(t\right)=\left\{\begin{array}{ccc} \frac{1}{\sqrt{T}} & for & t\in [0,T)\\ 0 & for & t\notin [0,T) \end{array}\right.$$

The real transmitted chirp becomes

(A8) $$s_{real}\left(t\right)=s\left(t\right)\cdot p_{T}\left(t\right)=A\cdot e^{j2\pi \cdot \left(\pm \frac{\left|\Delta f\right|}{2T}\cdot t^{2}+f_{0}\cdot t\right)}\cdot p_{T}\left(t\right)$$

(A9) $$s_{real}\left(t\right)=A\cdot e^{j2\pi \cdot \left(f_{0}\cdot t\pm \frac{1}{2}mt^{2}\right)}\cdot p_{T}\left(t\right)$$

## Appendix B. Derivation of the Ambiguity Function of the Linear Chirp Formula

The ambiguity function, $X(\tau ,f_{D})$, for a dual-linear chirp is derived as follows.

(A10) $$s\left(t\right)s^{*}\left(t-\tau \right)=A\cdot p_{T}\left(t\right)\cdot e^{j2\pi (f_{o}t+\frac{1}{2}mt^{2})}\cdot A^{*}\cdot p_{T}\left(t-\tau \right)\cdot e^{j2\pi (f_{o}\left(t-\tau \right)+\frac{1}{2}m{\left(t-\tau \right)}^{2})}$$

$$={\left|A\right|}^{2}\cdot p_{T}\left(t\right)\cdot p_{T}\left(t-\tau \right)\cdot e^{j2\pi (f_{o}t+\frac{1}{2}mt^{2})}\cdot e^{j2\pi (f_{o}\left(t-\tau \right)+\frac{1}{2}m{\left(t-\tau \right)}^{2})}$$

=|A|2·pT(t)·pT(t−τ)·ej2π[+fot+12mt2−fot+foτ−12m(t−τ)2]

=|A|2·pT(t)·pT(t−τ)·ej2π[foτ+12mt2−12mt2+mtτ−12mτ2]

(A11) $$s\left(t\right)s^{*}\left(t-\tau \right)={\left|A\right|}^{2}\cdot p_{T}\left(t\right)\cdot p_{T}\left(t-\tau \right)\cdot e^{j2\pi [f_{o}\tau +mt\tau -\frac{1}{2}m\tau^{2}]}$$

adding the Doppler term,

(A12) $$s\left(t\right)s^{*}\left(t-\tau \right)\cdot e^{-j2\pi f_{D}t}={\left|A\right|}^{2}\cdot p_{T}\left(t\right)\cdot p_{T}\left(t-\tau \right)\cdot e^{j2\pi [f_{o}\tau -\frac{1}{2}m\tau^{2}]}\cdot e^{j2\pi [\left(m\tau -f_{D}\right)t]}$$

integrating,

(A13) $$X\left(\tau ,f_{D}\right)=\int_{-\infty }^{\infty }{\left|A\right|}^{2}\cdot p_{T}\left(t\right)\cdot p_{T}\left(t-\tau \right)\cdot e^{j2\pi [f_{o}\tau -\frac{1}{2}m\tau^{2}]}\cdot e^{j2\pi [\left(m\tau -f_{D}\right)t]}dt$$

$$X\left(\tau ,f_{D}\right)={\left|A\right|}^{2}\cdot e^{j2\pi [f_{o}\tau -\frac{1}{2}m\tau^{2}]}\int_{-\infty }^{\infty }\cdot p_{T}\left(t\right)\cdot p_{T}\left(t-\tau \right)\cdot e^{j2\pi [\left(m\tau -f_{D}\right)t]}dt$$

$$X\left(\tau ,f_{D}\right)={\left|A\right|}^{2}\cdot e^{j2\pi [f_{o}\tau -\frac{1}{2}m\tau^{2}]}\cdot \frac{1}{T}\cdot \int_{a}^{b}e^{j2\pi [\left(m\tau -f_{D}\right)t]}dt$$

with a=max(0,τ), b=min(T,T+τ). The defined integral can be expressed as the Fourier transform of a displaced pulse in time,

$$\int_{a}^{b}e^{j2\pi ft}=\int_{-\infty }^{\infty }rect\left(\frac{t-\frac{a+b}{2}}{T}\right)\cdot e^{j2\pi ft}dt=F\left\{rect\left(\frac{t-\frac{a+b}{2}}{T}\right)\right\}=$$

$$=F\left\{rect\left(\frac{t}{T}\right)\right\}\cdot e^{-j2\pi \frac{a+b}{2}f}=T\cdot sinc\left(fT\right)\cdot e^{-j2\pi \frac{a+b}{2}f}$$

where T is of length L=b−a=T−|τ| and $\frac{a+b}{2}=\frac{T+\tau }{2}$. Resulting in,

$$X\left(\tau ,f_{D}\right)={\left|A\right|}^{2}\cdot \frac{1}{T}\cdot e^{j2\pi [f_{o}\tau -\frac{1}{2}m\tau^{2}]}\cdot e^{-j2\pi \left[\frac{a+b}{2}\right]\left[m\tau -f_{D}\right]}\cdot \left[L\right]\cdot sinc\left(\left[m\tau -f_{D}\right]\left[L\right]\right)$$

$$X\left(\tau ,f_{D}\right)={\left|A\right|}^{2}\cdot \frac{1}{T}\cdot e^{j2\pi [f_{o}\tau -\frac{1}{2}m\tau^{2}]}\cdot e^{-j2\pi \left[\frac{T+\tau }{2}\right]\left[m\tau -f_{D}\right]}\cdot [T-|\tau |]\cdot sinc(\left[m\tau -f_{D}\right]\left[T-|\tau |]\right)$$

(A14) $$X\left(\tau ,f_{D}\right)={\left|A\right|}^{2}\cdot e^{j2\pi [f_{o}\tau -\frac{1}{2}m\tau^{2}]}\cdot e^{-j2\pi \left[\frac{T+\tau }{2}\right]\left[m\tau -f_{D}\right]}\cdot \left[1-\frac{\left|\tau \right|}{T}\right]\cdot \frac{sin(\pi T\left[m\tau -f_{D}\right]\left[1-\frac{\left|\tau \right|}{T}\right])}{\pi T\left[m\tau -f_{D}\right]\left[1-\frac{\left|\tau \right|}{T}\right]}$$

(A15) $$|X\left(\tau ,f_{D}\right){\left|=|A\right|}^{2}\cdot \left[1-\frac{\left|\tau \right|}{T}\right]\cdot \left|\frac{sin(\pi T\left[m\tau -f_{D}\right]\left[1-\frac{\left|\tau \right|}{T}\right])}{\pi T\left[m\tau -f_{D}\right]\left[1-\frac{\left|\tau \right|}{T}\right]}\right|$$

## Appendix C. Derivation of the Ambiguity Function of the Dual-Linear Chirp

The ambiguity function, $X(\tau ,f_{D})$, for the dual-linear chirp with linearity, from Equation (A28), of integrals appears as

(A16) $$X\left(\tau ,f_{D}\right)=X_{↑↑}\left(\tau ,f_{D}\right)+X_{↓↓}\left(\tau ,f_{D}\right)+X_{↑↓}\left(\tau ,f_{D}\right)+X_{↓↑}\left(\tau ,f_{D}\right)$$

From the derivation made in Appendix B, $X_{↑↑}\left(\tau ,f_{D}\right)$ is the ambiguity function of an up-linear chirp and, $X_{↓↓}\left(\tau ,f_{D}\right)=X_{↑↑}\left(\tau ,f_{D}\right){|}_{m=-m}$, is the ambiguity function of a down-linear chirp. The only change when compared to the up-chirp is the slope’s sign, as derived in Appendix A. That is,

(A17) $$X_{↑↑}\left(\tau ,f_{D}\right)={\left|A\right|}^{2}\cdot e^{j2\pi [f_{o}\tau -\frac{1}{2}m\tau^{2}]}\cdot e^{-j2\pi \left[\frac{T+\tau }{2}\right]\left[m\tau -f_{D}\right]}\cdot \left[1-\frac{\left|\tau \right|}{T}\right]\cdot \frac{sin(\pi T\left[m\tau -f_{D}\right]\left[1-\frac{\left|\tau \right|}{T}\right])}{\pi T\left[m\tau -f_{D}\right]\left[1-\frac{\left|\tau \right|}{T}\right]}$$

(A18) $$|X_{↑↑}\left(\tau ,f_{D}\right){\left|=|A\right|}^{2}\cdot \left[1-\frac{\left|\tau \right|}{T}\right]\cdot \left|\frac{sin(\pi T\left[m\tau -f_{D}\right]\left[1-\frac{\left|\tau \right|}{T}\right])}{\pi T\left[m\tau -f_{D}\right]\left[1-\frac{\left|\tau \right|}{T}\right]}\right|$$

(A19) $$X_{↓↓}\left(\tau ,f_{D}\right)={\left|A\right|}^{2}\cdot e^{j2\pi [f_{o}\tau +\frac{1}{2}m\tau^{2}]}\cdot e^{-j2\pi \left[\frac{T+\tau }{2}\right]\left[-m\tau -f_{D}\right]}\cdot \left[1-\frac{\left|\tau \right|}{T}\right]\cdot \frac{sin(\pi T\left[-m\tau -f_{D}\right]\left[1-\frac{\left|\tau \right|}{T}\right])}{\pi T\left[-m\tau -f_{D}\right]\left[1-\frac{\left|\tau \right|}{T}\right]}$$

(A20) $$|X_{↓↓}\left(\tau ,f_{D}\right){\left|=|A\right|}^{2}\cdot \left[1-\frac{\left|\tau \right|}{T}\right]\cdot \left|\frac{sin(\pi T\left[-m\tau -f_{D}\right]\left[1-\frac{\left|\tau \right|}{T}\right])}{\pi T\left[-m\tau -f_{D}\right]\left[1-\frac{\left|\tau \right|}{T}\right]}\right|$$

As for the cross-terms, they develop as follows.

(A21) $$s_{↑}\left(t\right)s_{↓}^{*}\left(t-\tau \right)=A_{↑}\cdot p_{T}\left(t\right)\cdot e^{j2\pi [f_{o}t+\frac{1}{2}mt^{2}]}\cdot A_{↓}^{*}\cdot p_{T}\left(t-\tau \right)\cdot e^{-j2\pi [f_{o}\left(t-\tau \right)-\frac{1}{2}m{\left(t-\tau \right)}^{2}]}$$

=A↑·A↓*·pT(t)·pT(t−τ)·ej2π[+fot−fot+foτ+12mt2+12mt2+12mτ2−mtτ]

adding the Doppler term,

(A22) $$s_{↑}\left(t\right)s_{↓}^{*}\left(t-\tau \right)\cdot e^{-j2\pi f_{D}t}=A_{↑}\cdot A_{↓}^{*}\cdot p_{T}\left(t\right)\cdot p_{T}\left(t-\tau \right)\cdot e^{j2\pi [mt^{2}-mt\tau +f_{o}\tau +\frac{1}{2}m\tau^{2}]}\cdot e^{-j2\pi f_{D}t}$$

$$=A_{↑}\cdot A_{↓}^{*}\cdot p_{T}\left(t\right)\cdot p_{T}\left(t-\tau \right)\cdot e^{j2\pi [mt^{2}+\left(-m\tau -f_{D}\right)t+\left(\frac{1}{2}m\tau^{2}+f_{o}\tau \right)]}$$

$$=A_{↑}\cdot A_{↓}^{*}\cdot e^{j2\pi [f_{o}\tau +\frac{1}{2}m\tau^{2}]}\cdot p_{T}\left(t\right)\cdot p_{T}\left(t-\tau \right)\cdot e^{-j2\pi [mt^{2}+\left(-m\tau -f_{D}\right)t]}$$

rearranging the quadratic time terms by completing the square,

$$=A_{↑}\cdot A_{↓}^{*}\cdot e^{j2\pi [f_{o}\tau +\frac{1}{2}m\tau^{2}]}\cdot p_{T}\left(t\right)\cdot p_{T}\left(t-\tau \right)\cdot e^{j2\pi [m{\left(t+\frac{-m\tau -f_{D}}{2m}\right)}^{2}-\frac{{\left(-m\tau -f_{D}\right)}^{2}}{4m}]}$$

integrating,

(A23) $$X_{↑↓}\left(\tau ,f_{D}\right)=\int_{\infty }^{\infty }A_{↑}\cdot A_{↓}^{*}\cdot e^{j2\pi [f_{o}\tau +\frac{1}{2}m\tau^{2}]}\cdot p_{T}\left(t\right)\cdot p_{T}\left(t-\tau \right)\cdot e^{j2\pi [m{\left(t+\frac{-m\tau -f_{D}}{2m}\right)}^{2}-\frac{{\left(-m\tau -f_{D}\right)}^{2}}{4m}]}dt$$

$$=A_{↑}\cdot A_{↓}^{*}\cdot e^{j2\pi [f_{o}\tau +\frac{1}{2}m\tau^{2}]}\cdot e^{-j2\pi [\frac{{\left(-m\tau -f_{D}\right)}^{2}}{4m}]}\int_{a}^{b}e^{j2\pi [m{\left(t+\frac{-m\tau -f_{D}}{2m}\right)}^{2}]}dt$$

with a and b, as defined in Appendix B, and let $u=\sqrt{2m}\left(t-\frac{m\tau +f_{D}}{2m}\right)$ and $dt=\frac{du}{\sqrt{2m}}$

=A↑·A↓*·ej2π[foτ+12mτ2]·e−j2π[(−mτ−fD)24m]∫u(a)u(b)ej2π[u22]12mdu

$$=A_{↑}\cdot A_{↓}^{*}\cdot e^{j2\pi [f_{o}\tau +\frac{1}{2}m\tau^{2}]}\cdot e^{-j2\pi [\frac{{\left(-m\tau -f_{D}\right)}^{2}}{4m}]}\cdot \frac{1}{\sqrt{2m}}\cdot \int_{u(a)}^{u(b)}e^{j\pi u^{2}}du$$

(A24) $$X_{↑↓}\left(\tau ,f_{D}\right)=A_{↑}\cdot A_{↓}^{*}\cdot e^{j2\pi [f_{o}\tau +\frac{1}{2}m\tau^{2}]}\cdot e^{-j2\pi [\frac{{\left(-m\tau -f_{D}\right)}^{2}}{4m}]}\cdot \frac{1}{\sqrt{2m}}\cdot {\left[Fr(u)\right]}_{u(a)}^{u(b)}$$

where, Fr(u) is the complex Fresnel integral with u(t), t∈{a(τ),b(τ)}. Analogously, the other cross term,

(A25) $$X_{↓↑}\left(\tau ,f_{D}\right)=A_{↓}\cdot A_{↑}^{*}\cdot e^{j2\pi [f_{o}\tau -\frac{1}{2}m\tau^{2}]}\cdot e^{j2\pi [\frac{{\left(m\tau -f_{D}\right)}^{2}}{4m}]}\cdot \frac{1}{\sqrt{2m}}\cdot {\left[Fr(v)\right]}_{v(a)}^{v(b)}$$

with, Fr(v) being the complex Fresnel integral with $v\left(t\right)=\sqrt{2m}\left(t-\frac{m\tau -f_{D}}{2m}\right)$ (different quadratic center), for t∈{a(τ),b(τ)}. Finally, if the energy is equally distributed between up-down chirps,

(A26) $$X_{↑↓}\left(\tau ,f_{D}\right)={\left|A\right|}^{2}\cdot e^{j2\pi [f_{o}\tau +\frac{1}{2}m\tau^{2}]}\cdot e^{-j2\pi [\frac{{\left(-m\tau -f_{D}\right)}^{2}}{4m}]}\cdot \frac{1}{\sqrt{2m}}\cdot {\left[Fr(u)\right]}_{u(a)}^{u(b)}$$

(A27) $$X_{↓↑}\left(\tau ,f_{D}\right)={\left|A\right|}^{2}\cdot e^{j2\pi [f_{o}\tau -\frac{1}{2}m\tau^{2}]}\cdot e^{j2\pi [\frac{{\left(m\tau -f_{D}\right)}^{2}}{4m}]}\cdot \frac{1}{\sqrt{2m}}\cdot {\left[Fr(v)\right]}_{v(a)}^{v(b)}$$

### Integral Linearity

(A28)
$$X\left(\tau ,f_{D}\right)=\int_{\infty }^{\infty }s_{D}\left(t\right)s_{D}^{*}\left(t-\tau \right)\cdot e^{-j2\pi f_{D}t}dt=$$

$$=\int_{\infty }^{\infty }\left[s_{↑}\left(t\right)+s_{↓}\left(t\right)\right]{\left[s_{↑}\left(t-\tau \right)+s_{↓}\left(t-\tau \right)\right]}^{*}\cdot e^{-j2\pi f_{D}t}dt=$$

$$=\int_{\infty }^{\infty }\left[s_{↑}\left(t\right)+s_{↓}\left(t\right)\right]\left[s_{↑}^{*}\left(t-\tau \right)+s_{↓}^{*}\left(t-\tau \right)\right]\cdot e^{-j2\pi f_{D}t}dt=$$

$$=\int_{\infty }^{\infty }\left[s_{↑}\left(t\right)s_{↑}^{*}\left(t-\tau \right)+s_{↓}\left(t\right)s_{↓}^{*}\left(t-\tau \right)+s_{↑}\left(t\right)s_{↓}^{*}\left(t-\tau \right)+s_{↓}\left(t\right)s_{↑}^{*}\left(t-\tau \right)\right]\cdot e^{-j2\pi f_{D}t}dt=$$

$$=\int_{\infty }^{\infty }s_{↑}\left(t\right)s_{↑}^{*}\left(t-\tau \right)\cdot e^{-j2\pi f_{D}t}dt+\int_{\infty }^{\infty }s_{↓}\left(t\right)s_{↓}^{*}\left(t-\tau \right)\cdot e^{-j2\pi f_{D}t}dt$$

$$+\int_{\infty }^{\infty }s_{↑}\left(t\right)s_{↓}^{*}\left(t-\tau \right)\cdot e^{-j2\pi f_{D}t}dt+\int_{\infty }^{\infty }s_{↓}\left(t\right)s_{↑}^{*}\left(t-\tau \right)\cdot e^{-j2\pi f_{D}t}dt=$$

$$=X_{↑↑}\left(\tau ,f_{D}\right)+X_{↓↓}\left(\tau ,f_{D}\right)+X_{↑↓}\left(\tau ,f_{D}\right)+X_{↓↑}\left(\tau ,f_{D}\right)$$

## Appendix D. RMS Time Delay Error of an LFM Waveform

To estimate the error of a measurement, the difference between the true and measured values can be calculated with RMS. The bound for the RMS error in measuring the two-way time delay Tr=2Rc when using a LFM waveform, as proposed by Skolnik in [20] and ([19], Chapter 11) is

(A29) (ΔT)2¯12=tr(2SN)12=δT=1β2·E/N0

where $t_{r}$ is the signal slope or rise time, and may be approximated as 1β, the parameter β is the effective bandwidth and is 2π times the RMS deviation of the energy spectrum $\beta =2\pi \cdot \sigma_{f}$, or the angular RMS bandwidth, which is squared

(A30) $$\beta^{2}=\frac{{\left(2\pi \right)}^{2}\int_{-\infty }^{\infty }{\left(f-\mu_{f}\right)}^{2}{\left|S\left(f\right)\right|}^{2}df}{\int_{-\infty }^{\infty }{\left|S\left(f\right)\right|}^{2}df}$$

where $\mu_{f}$ is the first order statistic referred to as the mean, $\mu_{f}=\frac{1}{E}\int_{-\infty }^{\infty }f\cdot {\left|S\left(f\right)\right|}^{2}df$.

Using the approximation proposed by Cook in [34], a LFM with a large time-bandwidth product that approaches a rectangular spectrum may be written as

(A31) $${\left|S\left(f\right)\right|}^{2}=\left\{\begin{array}{ccc} C & for & f\in [f_{0},f_{1})\\ 0 & for & f\notin [f_{0},f_{1}) \end{array}\right.$$

the RMS deviation is

(A32) $$\sigma_{f}^{2}=\frac{\int_{-\infty }^{\infty }{\left(f-\mu_{f}\right)}^{2}{\left|S\left(f\right)\right|}^{2}df}{\int_{-\infty }^{\infty }{\left|S\left(f\right)\right|}^{2}df}$$

$$=\frac{\int_{-\infty }^{\infty }\left(f^{2}-2\mu_{f}\cdot f+\mu_{f}^{2}\right){\left|S\left(f\right)\right|}^{2}df}{E}=\frac{1}{E}\int_{-\infty }^{\infty }\left(f^{2}-2\mu_{f}\cdot f+\mu_{f}^{2}\right){\left|S\left(f\right)\right|}^{2}df=$$

$$=\frac{1}{E}\left(\int_{-\infty }^{\infty }f^{2}{\left|S\left(f\right)\right|}^{2}df-2\mu_{f}\cdot \int_{-\infty }^{\infty }{f|S\left(f\right)|}^{2}df+\mu_{f}^{2}\int_{-\infty }^{\infty }{\left|S\left(f\right)\right|}^{2}df\right)=$$

$$=\frac{1}{E}\int_{-\infty }^{\infty }f^{2}{\left|S\left(f\right)\right|}^{2}df-2\mu_{f}\cdot \frac{1}{E}\int_{-\infty }^{\infty }{f|S\left(f\right)|}^{2}df+\mu_{f}^{2}\cdot \frac{1}{E}\int_{-\infty }^{\infty }{\left|S\left(f\right)\right|}^{2}df$$

$$=\frac{1}{E}\int_{-\infty }^{\infty }f^{2}{\left|S\left(f\right)\right|}^{2}df-2\mu_{f}\cdot \mu_{f}+\mu_{f}^{2}\cdot \frac{1}{E}\cdot E=$$

(A33) $$\sigma_{f}^{2}=\frac{1}{E}\int_{-\infty }^{\infty }f^{2}{\left|S\left(f\right)\right|}^{2}df-\mu_{f}^{2}$$

with the signal energy being

$$E=\int_{\infty }^{\infty }{\left|S\left(f\right)\right|}^{2}df=\int_{f_{0}}^{f_{1}}Cdf=C\cdot \left[f_{1}-f_{0}\right]=C\cdot B$$

the mean is

$$\mu_{f}^{2}=\frac{1}{E}\int_{f_{0}}^{f_{1}}f\cdot C\;df=\frac{1}{CB}\cdot C\cdot {\left[\frac{f^{2}}{2}\right]}_{f_{0}}^{f_{1}}=\frac{1}{B}\cdot \frac{\left(f_{1}-f_{0}\right)\left(f_{1}+f_{0}\right)}{2}=\frac{f_{1}+f_{0}}{2}\equiv f_{c}$$

the variance is calculated such

$$\sigma_{f}^{2}=\frac{1}{CB}\cdot \int_{f_{0}}^{f_{1}}f^{2}\cdot C\;df-f_{c}^{2}=\frac{1}{B}\cdot {\left[\frac{f^{3}}{3}\right]}_{f_{0}}^{f_{1}}-f_{c}^{2}=\frac{1}{B}\cdot \left[\frac{f_{1}^{3}-f_{0}^{3}}{3}\right]-f_{C}^{2}$$

$$=\frac{1}{B}\cdot \left[\frac{\left(f_{1}-f_{0}\right)\left(f_{1}^{2}+f_{1}f_{0}+f_{0}\right)}{3}\right]-f_{C}^{2}=$$

$$=\left[\frac{f_{1}^{2}+f_{1}f_{0}+f_{0}^{2}}{3}\right]-f_{C}^{2}=\left[\frac{\left(f_{c}^{2}+f_{c}B+\frac{B^{2}}{4}\right)+\left(f_{c}^{2}+\frac{B^{2}}{4}\right)+\left(f_{c}^{2}-f_{c}B+\frac{B^{2}}{4}\right)}{3}\right]-f_{C}^{2}$$

(A34) $$\sigma_{f}^{2}=\left[\frac{3\cdot f_{c}^{2}+\frac{B^{2}}{4}}{3}\right]-f_{c}^{2}=\frac{B^{2}}{12}$$

the squared angular RMS bandwidth is

(A35) $$\beta^{2}={\left(2\pi \right)}^{2}\cdot \sigma_{f}^{2}={\left(2\pi \right)}^{2}\cdot \frac{B^{2}}{12}$$

thus,

(A36) $$\beta =\left(2\pi \right)\cdot \frac{B}{\sqrt{12}}=\frac{\pi B}{\sqrt{3}}$$

the RMS time delay error for a LFM pulse is

(A37) $$\delta T_{r}=\frac{1}{\frac{\pi B}{\sqrt{3}}\sqrt{2\cdot E/N_{0}}}=\frac{\sqrt{3}}{\pi B\sqrt{2\cdot E/N_{0}}}$$

and the range error is

(A38) δR=c·δT

## Appendix E. World Geodetic System 84 and Earth Gravitation Model 96

The World Geodetic System 1984 (WGS84) is a reference system intended for the measurement and mapping of the Earth’s surface. It is commonly used among other applications, such as for GNSS or propagation of orbital parameters. It models the Earth’s surface with a reference ellipsoid, providing a geodetic reference. It is often combined with Earth Gravitational Model (EGM)96 which models a geoid of height *N* at a specific latitude. It approximates the mean sea level by taking into account the gravity field of the Earth.

For geometrical calculations, the reference ellipsoid is used. It represents the distance from Earth’s center of mass to the surface of the ellipsoid *h* at a specific latitude. It is calculated as follows:

(A39) $$R_{EARTH}=\sqrt{\frac{{\left[a^{2}\cdot \cos\left(\phi_{gs}\right)\right]}^{2}+{\left[b^{2}\cdot \sin\left(\phi_{gs}\right)\right]}^{2}}{{\left[a\cdot \cos\left(\phi_{gs}\right)\right]}^{2}+{\left[b\cdot \sin\left(\phi_{gs}\right)\right]}^{2}}}$$

where $\phi_{gs}$ is the latitude, *a* is the equatorial radius, i.e., semi-major axis, of value 6378.137 km, and *b* is the polar radius, i.e., semi-minor axis, of value 6356.752 km.

Note, however, that a point on the Earth’s surface with geodetic latitude and longitude, ϕ and λ, will be calculated as,

(A40) $$r\left(\phi ,h\right)=R_{EARTH}\left(\phi \right)+\Delta \left(\phi ,h\right)$$

which is dependent on *h* but not directly on $R_{EARTH}\left(\phi \right)+h$ since one is defined along the ellipsoid normal and the other along the radial direction. Δ(ϕ,h) is an additional offset due to the projection of the ellipsoidal height onto the radial direction. It coincides only at the equator and poles.

The orthometric height is from the Earth’s surface at a specific latitude and the geoid. The relation between the three magnitudes is approximated by

(A41) H=h−N[m]

where *H* is the orthometric height, *h* is the ellipsoidal height and *N* is the geoid height.

## Appendix F. Differences Between the Time Standardized SATRE-Based TWSTFT and the Proposed Measurement Method

Current TWSTFT implementations are standardized and follow the ITU-R TF.1153-4 recommendation [36]. The TWSTFT is made throughout the transponder of specific GEOs satellites with an allocated bandwidth for the purpose. The participating stations use code division multiple access (CDMA) to access the channel and compare their respective LOs. Each station encodes the PPS from its LO with BPSK ([49], Chapter 6).

The different versions of the TWSTFT implementation have been, in their time, the state of the art for high-accuracy intercontinental time comparison. TWSTFT plays a fundamental role in the calculation and maintenance the UTC reference time scale. Such implementations profit from first-order geometry path cancellation by sending pseudo-random-noise (PRN)-modulated signals produced and processed by dedicated hardware modems such as the SATRE family.

Despite the maturity of conventional TWSTFT, the current version finds as a major limitation the diurnal variations during a measurement [40,50]. Building upon this work, SDR mixed with the SATRE modem were introduced, replacing proprietary hardware correlators of the conventional system with digital signal processing, while preserving the standardized part of the system such as the PRN spreading code and two-way measurement principle. Such an extension reduced systematic noise and diurnals by a factor of 2 or 3 in short continental links, as well as highlighting the need of a pure SDR alternative to the modem techniques for TWSTFT [16].

In Table A1, the main differences of the aforementioned liaison between conventional TWSTFT and the proposed method are listed.

**Table A1:** Characteristic comparison between SATRE-based TWSTFT and the proposed measurement method.

Characteristic	SATRE-Based TWSTFT	SDR-Based Radar Ranging (Proposed Method)
**Principle and Standardization**		
measurement principle	Two-way time transfer	Loop back ranging using matched filtering and pulse compression
Standardization	Fully standardized (ITU, BIPM, CCTF)	Non-standard, experimental
difference with UTC time scale	comparable	Non-comparable without additional framework
**Transmitted Signal**		
Signal type	Continuous PRN-modulated carrier, spread by chip rate	Burst of linear FM chirps per measurement
Effective bandwidth	1–2.5 MHz (fixed)	Variable, *B* (user-defined)
Duty cycle	Near 100% during a session	Configurable
**Received Signal Processing**		
Correlation method	PRN code correlator	Matched filtering (FFT-based)
**Time Structure of One Measurement**		
Measurement duration	Typically 300 s session	User-defined (ms to seconds)
Internal averaging	$10^{8}$–$10^{9}$ PRN chips per session	*N* pulses or chirps
**Noise, Averaging, and Scaling**		
Dominant short-term noise	Code correlation noise	Thermal noise, ADC jitter, clock phase noise
Timing resolution scaling	Limited by chip rate	$\sigma_{\tau }\propto \frac{1}{B\sqrt{\mathrm{SNR}}}$
Effect of increasing bandwidth	Not applicable	Improvement of timing precision
Averaging behavior	Limited by diurnals	$\propto 1/\sqrt{N}$ for *N* pulses
